# Evolution and diversification of the momilactone biosynthetic gene cluster in the genus *Oryza*

**DOI:** 10.1111/nph.20416

**Published:** 2025-01-30

**Authors:** Santiago Priego‐Cubero, Youming Liu, Tomonobu Toyomasu, Michael Gigl, Yuto Hasegawa, Hideaki Nojiri, Corinna Dawid, Kazunori Okada, Claude Becker

**Affiliations:** ^1^ Faculty of Biology Ludwig‐Maximilians‐Universität München 82152 Martinsried Germany; ^2^ Agro‐Biotechnology Research Center (AgTECH), Graduate School of Agricultural and Life Sciences (GSALS) The University of Tokyo Tokyo 113‐8657 Japan; ^3^ Faculty of Agriculture Yamagata University Tsuruoka Yamagata 997‐8555 Japan; ^4^ Professorship for Functional Phytometabolomics, TUM School of Life Sciences Technical University of Munich Lise‐Meitner‐Str. 34 85354 Freising Germany

**Keywords:** biosynthetic gene cluster, *Echinochloa crus‐galli*, gene cluster evolution, momilactone, *Oryza*, phytoalexins

## Abstract

- Plants are master chemists and collectively are able to produce hundreds of thousands of different organic compounds. The genes underlying the biosynthesis of many specialized metabolites are organized in biosynthetic gene clusters (BGCs), which is hypothesized to ensure their faithful coinheritance and to facilitate their coordinated expression. In rice (*Oryza sativa*), momilactones are diterpenoids that act in plant defence and various organismic interactions. Many of the genes essential for momilactone biosynthesis are grouped in a BGC.
- We applied comparative genomics of diploid and allotetraploid *Oryza* species to reconstruct the species‐specific architecture, evolutionary trajectory, and sub‐functionalisation of the momilactone biosynthetic gene cluster (MBGC) in the *Oryza* genus.
- Our data show that the evolution of the MBGC is marked by lineage‐specific rearrangements and gene copy number variation, as well as by occasional cluster loss. We identified a distinct cluster architecture in *Oryza coarctata*, which represents the first instance of an alternative architecture of the MBGC in *Oryza* and strengthens the idea of a common origin of the cluster in *Oryza* and the distantly related genus *Echinochloa*.
- Our research illustrates the evolutionary and functional dynamics of a biosynthetic gene cluster within a plant genus.

## Introduction

### Biosynthetic gene clusters and their evolution

With more and more plant reference genome assemblies becoming available, biosynthetic gene clusters (BGCs), i.e., the co‐localization of often phylogenetically unrelated genes that participate in the same biosynthetic cascade of specialized metabolites, have emerged as a common feature of genomic organization in plants (Polturak *et al*., 2022b). BGCs are postulated to confer evolutionary advantages because they facilitate coordinated gene expression, enable the reliable coinheritance of genes involved in the same metabolic pathway (thereby preventing the accumulation of toxic intermediates), or facilitate the formation of metabolons (Nützmann *et al*., 2016). However, the mechanisms by which such nonorthologous genes become localized in the same genomic region and act in the same biosynthetic pathway are still poorly understood. Currently, the most common model proposes that they have formed through a series of events that is driven by both positive‐ and negative‐selection pressure, starting with gene duplication, followed by neofunctionalization, and ultimately relocation. In some cases, this process appears to have been mediated by transposable elements (Polturak *et al*., 2022b; Smit & Lichman, 2022).

### Biological functions of rice phytoalexins, labdane‐related diterpenoids and momilactones

Phytoalexins are low‐molecular‐mass specialized plant metabolites that are often produced under biotic and abiotic stress conditions (Ahuja *et al*., 2012). In rice (*Oryza sativa*), the major phytoalexins are a group of labdane‐related diterpenoids (reviewed in Toyomasu *et al*., 2020), which derive from the cyclization of geranylgeranyl diphosphate (GGPP) into *ent*, *syn*, or normal stereoisomers of copalyl diphosphate (CDP) by the class II diterpene synthases Copalyl Diphosphate Synthases (CPSs). The biosynthesis of these metabolites has evolved from that of gibberellins (GAs), *ent* labdane‐related diterpenoids themselves, through duplication and neofunctionalization of core biosynthetic enzymes (Zi *et al*., 2014). Several *ent* and *syn* (but not normal) rice labdane‐related diterpenoids have been identified, including momilactones A and B, phytocassanes A to F, and oryzalexins (A to F, and S) (Zi *et al*., 2014; Toyomasu *et al*., 2020). Notably, momilactone A and, more prominently, momilactone B have a strong allelopathic activity, that is they inhibit the germination and growth of nearby plants upon being released by the rice plants into the soil (Kato *et al*., 1973; Kato‐Noguchi *et al*., 2010; Serra Serra *et al*., 2021). Both compounds accumulate in rice husks but are also exuded from the roots (Kato‐Noguchi & Ino, 2003; Kato‐Noguchi *et al*., 2010).

### Biosynthesis of momilactones and clustering of momilactone genes

**Fig. 1:**
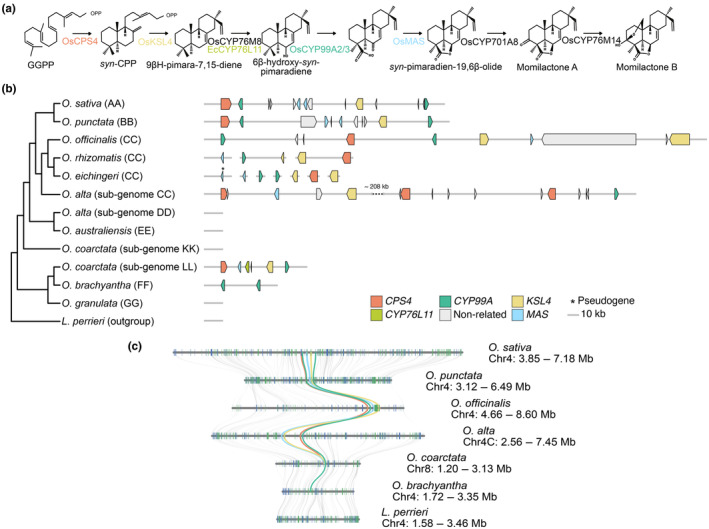
The momilactone biosynthetic gene cluster in the *Oryza* genus. (a) Simplified representation of the momilactone biosynthetic pathway, adapted from De La Peña & Sattely (2021) and Kitaoka *et al*. (2021). (b) Phylogenetic relationship between the different *Oryza* species and sub‐genomes included in this study, the outgroup *Leersia perrieri*, and their respective MBGCs. The species tree represents the maximum‐likelihood tree inferred from a concatenated multiple‐sequence alignment of single‐copy orthologues using 4069 orthogroups with a minimum of 100.0% of species having single‐copy genes in any orthogroup. Coloured block arrows represent genes. Scaffold and positional information on all genes is provided in Table S2. (c) Microsynteny between the genomic regions containing the MBGC from *Oryza sativa*, *Oryza punctata*, *Oryza officinalis*, *Oryza alta*, *Oryza coarctata*, *Oryza brachyantha*, and *L. perrieri*. In (c), grey lines connect the respective orthologues. ‘Nonrelated’ refers to genes that do not seem to be functionally related to the main biosynthetic cascade of the cluster.

Momilactone biosynthesis (Fig. 1a) starts with the cyclization of GGPP into *syn*‐copalyl diphosphate (*syn*‐CDP), catalysed by the Copalyl Diphosphate Synthase 4 (CPS4) (Otomo *et al*., 2004b; Xu *et al*., 2004). *syn*‐CDP is further cyclized into 9βH‐pimara‐7,15‐diene (also known as *syn*‐pimaradiene in the literature) by *ent*‐kaurene synthase‐like 4 (KSL4), a class I diterpene synthase (Otomo *et al*., 2004a; Wilderman *et al*., 2004). Because *syn*‐CDP is also a substrate for oryzalexin S biosynthesis, the KSL4‐mediated cyclization is the first truly dedicated step towards momilactone production (Tamogani *et al*., 1993). 9βH‐pimara‐7,15‐diene undergoes several oxidation steps, catalysed first by cytochrome P450 (CYP) monooxygenases CYP76M8 and CYP99A3, followed by the short‐chain alcohol dehydrogenase MOMILACTONE A SYNTHASE (MAS) and CYP701A8, to yield momilactone A (Fig. 1a) (De La Peña & Sattely, 2021; Kitaoka *et al*., 2021). CYP76M14 catalyses the final hydroxylation of C20, leading to spontaneous closure of the hemi‐acetal ring and forming momilactone B (De La Peña & Sattely, 2021). Among the biosynthetic genes, *CPS4*, *KSL4*, and the paralogs *CYP99A2/3* and *MAS1/2* are co‐localized on chromosome 4 in a momilactone biosynthetic gene cluster (MBGC) (Shimura *et al*., 2007; Miyamoto *et al*., 2016). However, not all genes required for momilactone biosynthesis are contained in the MBGC: *CYP76M8* is located on chromosome 2 and is part of another BGC that is required for phytocassane and oryzalexin production (Okada, 2011; Kitaoka *et al*., 2021), while *CYP701A8* and *CYP76M14* are located on chromosomes 6 and 1, respectively (De La Peña & Sattely, 2021).

### Evolution of the momilactone biosynthetic gene cluster in *Oryza*

The *Oryza* genus (belonging to the *Poaceae* family) consists of 27 known species with 11 different genome types (classified based on cytogenetics and genetic hybridization studies): 6 diploids (*n* = 12; AA, BB, CC, EE, FF and GG) and 5 allotetraploids (*n* = 24; BBCC, CCDD, HHJJ, HHKK and KKLL) (Ge *et al*., 1999; Lu *et al*., 2009). The MBGC was established in *Oryza* before the domestication of rice (*O. sativa*; AA) (Miyamoto *et al*., 2016). It is highly conserved among AA and BB genome type *Oryza* species, while only a partial cluster exists in the early‐divergent *Oryza* species *O. brachyantha* (FF), which harbours only two clustered *CYP99A2/3* paralogs (Miyamoto *et al*., 2016). Because the MBGC is incomplete in *O. brachyantha*, it likely evolved before the divergence of the BB lineage in *Oryza* (Miyamoto *et al*., 2016). Due to the limited availability of genome assemblies from species positioned between *O. brachyantha* and *O. punctata* in the phylogenetic tree, it has remained unclear whether the MBGC was restricted to species of the AA and BB lineages. Alternatively, the MBGC could have been lost specifically in the *O. brachyantha* lineage and might be present in other *Oryza* lineages. Lastly, the presence and configuration of the MBGC have not yet been studied in any of the allotetraploid *Oryza* species, and it therefore remains unknown if the cluster is present in both their sub‐genomes.

### Momilactone biosynthetic gene cluster in other species

Studies of the MBGC have not been limited to the *Oryza* genus; some have expanded into the wider *Poaceae* family. *Poaceae* encompass 12 subfamilies, of which nine belong to two core clades: BOP and PACMAD. The BOP clade comprises Bambusoideae, Oryzoideae (including *Oryza*), and Pooideae (including the *Triticeae* tribe); the PACMAD clade consists of Panicoideae, Aristidoideae, Chloridoideae, Micrairoideae, Arundinoideae, and Danthonioideae (Soreng *et al*., 2017). The MBGC is not restricted to the *Oryza* genus and has been identified through phylogenomic methods in species belonging to the PACMAD clade, specifically in the Panicoideae and Chloridoideae subfamilies (Wu *et al*., 2022a). However, it remains uncertain whether these species actually produce momilactones or other types of labdane‐related diterpenoids. *Echinochloa crus‐galli* (Panicoideae), a prevalent weed associated with rice cultivation, is the only species confirmed to produce momilactone A (Kraehmer *et al*., 2016; Wu *et al*., 2022a). Interestingly, *E. crus‐galli* exhibits a distinct MBGC architecture, including an additional cytochrome P450 (*EcCYP76L11*) (Guo *et al*., 2017; Kitaoka *et al*., 2021). EcCYP76L11, similarly to CYP76M8 in *Oryza sativa*, can catalyse the conversion of 9βH‐pimara‐7,15‐diene into 6β‐hydroxy‐*syn*‐pimaradiene (Kitaoka *et al*., 2021). Despite the shared origin of the MBGC in *Oryza* and *Echinochloa*, the *CYP76L11* gene has not been identified in the MBGC or the genome of any *Oryza* species (Wu *et al*., 2022a).

Here, we studied the presence, architecture, and evolution of the MBGC in cultivated rice and wild relatives from the *Oryza* genus by mining recently published genome assemblies. Our study shows that the MBGC is not restricted to species of the AA and BB lineages; we identify MBGC‐like clusters in *Oryza* CC species and in one of the respective sub‐genomes of the tetraploid species *O. alta* as well as in *O. coarctata*, a basal lineage in the *Oryza* phylogeny. We also show that the gene cluster was lost in several intermediate genome types. While momilactone A was detectable in all species harbouring the cluster, we were not able to detect momilactone B in *O. coarctata*, which might suggest that momilactone B production could have been a more recent innovation that emerged after the branching off of the KK genome type. In the *O. coarctata* MBGC, we identified an additional CYP that is different from the canonical *O. sativa* CYP99A2/3 and for which a corresponding orthologue could be found only in the MBGC of *E. crus‐galli*, altogether suggesting the existence of an early ancestral cluster. In summary, our study shows how a biosynthetic gene cluster diversified within a plant genus while largely maintaining its biosynthetic function.

## Materials and Methods

### *Oryza* and Poaceae datasets

*Oryza sativa* L. (v.7.0) (Ouyang *et al*., 2007) protein sequences and gene annotations were downloaded from Phytozome (https://phytozome‐next.jgi.doe.gov/); *Oryza punctata* Kotschy ex Steud. (Oryza_punctata_v1.2) (Stein *et al*., 2018) protein sequences and gene annotations from EnsemblPlants; the *Oryza officinalis* Wall. ex Watt (Shenton *et al*., 2020) genome sequence from NCBI (assembly GCA_008326285.1) and annotations from the original article repository; *Oryza eichingeri* Peter and *Oryza rhizomatis* Vaughan (Shenton *et al*., 2020) protein sequences and gene annotations from Cyverse Data Commons (doi: 10.25739/awh3‐dm39); *Oryza alta* Swallen (Yu *et al*., 2021) protein sequences and gene annotations from the from the Genome Warehouse (GWH) accession no. GWHAZTO00000000; the *Oryza australiensis* Domin (Phillips *et al*., 2022) genome sequence from the National Center for Biotechnology Information (NCBI) (GCA_019925245.2) and annotations from figShare (https://figshare.com/collections/Oryza_australiensis_Keep_River_Genome_Assembly_V2/5875592/2); the *Oryza coarctata* Roxb. (Zhao *et al*., 2023) protein sequences and gene annotation from figShare (https://figshare.com/articles/dataset/A_high‐quality_chromosome‐level_wild_rice_genome_of_i_Oryza_coarctata_i_/23938590/1); the *Oryza brachyantha* A.Chev. & Roehr. (Chen *et al*., 2013) (ObraRS2) genome assembly and gene annotations from NCBI (GCF_000231095.2); *Oryza granulata* Nees et Arn. ex Watt (Shi *et al*., 2020) protein sequences and gene annotations from GWH (GWHAAKB00000000); *Leersia perrieri* (A.Camus) Launert (Stein *et al*., 2018) protein sequences and gene annotations (v.1.4) from EnsemblPlants. *Oryza officinalis*, *Oryza australiensis* and *Oryza brachyantha* protein sequences were extracted from the gene annotation using gffread (v.0.12.7) (Pertea & Pertea, 2020).

*Aegilops tauschii* subsp. *strangulata* (Eig) Tzvelev (v.5.0; GCF_002575655.2) (Wang *et al*., 2021) protein sequences were retrieved from NCBI, *Brachypodium stacei* Catalán, Joch.Müll., L.A.J.Mur & T.Langdon (v.1.1) (Gordon *et al*., 2020) from Phytozome, *Brachypodium distachyon* L. (V.3.1) (Vogel *et al*., 2010; Sreedasyam *et al*., 2023) from Phytozome; *Cleistogenes songorica* Roshev. (Zhang *et al*., 2021) from GWH accession PRJCA002752; *Digitaria exilis* (Kippist) Stapf (CM05836) (Abrouk *et al*., 2020) from article repository in Dryad; *Eleusine coracana* L. (v.1.1) (Devos *et al*., 2023) from Phytozome; *Echinochloa crus‐galli* L. (v.3.0) (Wu *et al*., 2022b) sequences from GWH, accession no. GWHBDNR00000000; *Echinochloa haploclada* Stapf from (Ye *et al*., 2020) article repository; *Eragrostis curvula* (Schrad.) Nees (CERZOS_EC1.0; GCA_007726485.1) (Carballo *et al*., 2019) from NCBI; *Eragrostis tef* (Zuccagni) Trotter (ASM97063v1) (Cannarozzi *et al*., 2014) from Ensembl Plants; *Hordeum vulgare* L. cv Morex (V3) (Mascher *et al*., 2021) sequences from e!DAL (doi: 10.5447/ipk/2021/3), we specifically used the file Hv_Morex.pgsb.Jul2020.aa.fa that includes low confidence proteins. Unlike in databases such as Phytozome and EnsemblPlants, this annotation includes most of the gene in the diterpene cluster reported by (Liu *et al*., 2024); *Olyra latifolia* L. (Guo *et al*., 2019) sequences from http://www.genobank.org/bamboo; *Oropetium thomaeum* (v.1.0) (VanBuren *et al*., 2015) from Phytozome; *Phyllostachys edulis* (Carrière) J.Houz. (Zhao *et al*., 2018) from http://gigadb.org/dataset/100498; *Panicum hallii* var. *filipes* Vasey (V3.2) (Lovell *et al*., 2018) sequences from Phytozome; *Panicum virgatum* L. (v.5.1) (Lovell *et al*., 2021; Sreedasyam *et al*., 2023) from Phytozome; *Paspalum vaginatum* Sw. (v.3.1) (Sun *et al*., 2022) from Phytozome; *Pharus latifolius* L. (Ma *et al*., 2021) sequences from http://www.genobank.org/grass; *Sorghum bicolor* (L.) Moench (v.3.1.1) (McCormick *et al*., 2018) from Phytozome, *Secale cereale* L. (Lo7_2018v1p1p1.pgsb.Feb2019.HC) (Rabanus‐Wallace *et al*., 2021) from article repository; *Setaria italica* (L.) P.Beauv. (v.2.2) (Bennetzen *et al*., 2012) sequences from Phytozome; *Triticum aestivum* L. cv Chinese Spring (IWGSC V2.1) (Zhu *et al*., 2021) from https://urgi.versailles.inrae.fr/download/iwgsc/IWGSC_RefSeq_Annotations/v2.1/ (file iwgsc_refseqv2.1_annotation_200916_HC_pep.valid), *Zea mays* L. cv B73 (Schnable *et al*., 2009) (V4) from Phytozome, *Zizania palustris* L. (Haas *et al*., 2021) from NCBI (accession JAAALK000000000), and *Ananas comosus* (L.) Merr. (V3) (Ming *et al*., 2015) proteome from Phytozome as outgroup.

### MBGC orthologue detection

OrthoFinder (v.2.5.4) (Emms & Kelly, 2015, 2019) was used to infer orthogroups containing *O. sativa* momilactone biosynthesis genes (i.e. genes from multiple species descended from a single gene in the last common ancestor of these species). In the case of the *Oryza* dataset, the input for OrthoFinder included primary transcript proteomes of the following species: *O. sativa*, *O. punctata*, *O. officinalis*, *O. eichingeri*, *O. rhizomatis*, *O. alta*, *O. australiensis*, *O. coarctata*, *O. brachyantha*, *O. granulata*, and *L. perrieri* (included as the outgroup). We used the ‘‐M msa’ option to also infer the species tree (described in Emms & Kelly, 2018). Briefly, a maximum‐likelihood species tree was inferred by using concatenated multiple‐sequence alignment of single‐copy orthologues using 4069 orthogroups with a minimum of 100.0% of species having single‐copy genes in any orthogroup.

Each orthogroup was aligned using Mafft (v.7.490) (Katoh *et al*., 2002; Katoh & Standley, 2013). Iqtree (v.2.1.4‐beta) (Minh *et al*., 2020) was used to infer the maximum‐likelihood (ML) tree with the best‐fit model selected by ModelFinder (Kalyaanamoorthy *et al*., 2017) with 1000 replicates of ultrafast bootstrapping (Hoang *et al*., 2018).

Maximum‐likelihood rooted trees of each orthogroup were used to identify orthologues of *O. sativa* momilactone biosynthetic genes in *Oryza*. Genes within the same clade as *O. sativa* momilactone biosynthesis genes were considered as potentially clustered homologs. The genomic location upstream and downstream of these homologs was further analysed to confirm clustering. Trees were represented with Dendroscope (v.3.8.2).

As a quality control measure to ensure no potential momilactone genes were missed, we performed nBLAST using the genomic locus of each momilactone gene from *O. sativa* against each of the *Oryza* species studied. Where necessary, we manually annotated the given locus (i.e. *OoCYP76M14*).

### *Oryza coarctata* CYP76L11 orthologues

To infer the relationship between *EcCYP76L11 and OcoCYP76L11*, we used OrthoFinder to infer the orthogroup containing OcoCYP76L11. The input for OrthoFinder included the primary transcript proteomes of the *Oryza* species described previously, together with *Z. palustris* (Oryzoideae), *T. aestivum*, *A. tauschii*, *S. cereale*, *D. villosum*, *H. vulgare*, *B. distachyon*, *B. stacei* (Pooideae); *P. edulis*, *O. latifolia* (Bambusoideae); *Z. mays*, *S. bicolor*, *P. vaginatum*, *E. crus‐galli*, *E. haploclada*, *S. italica*, *P. hallii*, *P. virgatum*, *D. exilis* (Panicoideae); *E. coracana*, *C. songorica*, *O. thomaeum*, *E. curvula*, *E. tef* (Chloridoideae); *P. latifolius*, as outgroup to the core Poaceae; and *A. comosus* as outgroup of Poaceae.

The orthogroup containing OcoCYP76L11 was aligned using Mafft (v.7.490) followed by the inference of the maximum‐likelihood (ML) tree with Iqtree (v.2.1.4‐beta) and selecting the best‐fit model by ModelFinder with 1000 replicates of ultrafast bootstrapping.

### Microsynteny analysis

Microsynteny analysis and figures were done with MCScan implemented in Python (v.3.9) with JCVI utility libraries (v.1.1.11) following the package workflow: https://github.com/tanghaibao/jcvi/wiki/MCscan‐(Python‐version). Scripts are detailed in https://github.com/spriego/Priego‐Cubero‐et‐al.‐Oryza. Briefly, protein FASTA files and GFF3 files of each species were used as input. When needed, the FASTA files were processed using Biopython's seqio package (Cock *et al*., 2009). Pairwise orthologue and synteny blocks search was performed with default settings using a lift‐over distance of 5, a c‐score = 0.99 and a maximum of 1 iterations. Multi‐synteny blocks were constructed combining the syntenic blocks detected on every species with respect to *Oryza sativa*.

### RNA‐Seq mapping and quantification

Publicly available RNA‐Seq datasets from *O. officinalis* and *O. coarctata* were used (see Supporting Information Fig. S1). RNA‐Seq mapping and quantification was done using the nf‐core pipeline rnaseq v.3.14 (https://nf‐co.re/rnaseq/3.14.0) in Nextflow 22.10.4 (Patel *et al*., 2024). Briefly, adapter and quality trimming was performed using TrimGalore v.0.6.7 (Krueger *et al*., 2021). Then, reads were mapped to reference genomes using Star v.2.7.9a (Dobin *et al*., 2013) and quantified using salmon v.1.10.1 (Patro *et al*., 2017). Finally, feature counts were normalised by gene length and sequencing depth using transcripts per million (TPM), that were used to inspect the expression of the momilactones biosynthetic genes.

### SNP calling

Short‐read Illumina sequencing data for 15 *O. officinalis* accession were downloaded from OryzaGenome 2.1 (http://viewer.shigen.info/oryzagenome21detail/index.xhtml) (Kajiya‐Kanegae *et al*., 2021). SNP calling was performed using the nf‐core pipeline sarek v.3.0.1 with default parameters in Nextflow 22.10.4 (Cannarozzi *et al*., 2014; Ewels *et al*., 2020; Garcia *et al*., 2020). Briefly, the fastq files underwent sequence quality assessment and preprocessing using fastp v.0.23.2 (Chen *et al*., 2018). The reads were mapped to the *O. officinalis* reference genome using Bwa mem v.0.7.17‐r1188 (Katoh & Standley, 2013). Duplicates were marked with Gatk MarkDuplicates v.4.2.6.1 (McKenna *et al*., 2010). Variant calling was performed with HaplotypeCaller (Poplin *et al*., 2018). The BaseRecalibrator step was skipped as it requires a database of known polymorphic sites, which is unavailable for *O. officinalis*. The output vcf files were merged with bcftools v.1.13 (Danecek *et al*., 2021) and SNPs effect were annotated with snpEff v.5.0.1 (Cingolani *et al*., 2012).

### Data analysis

We used RStudio with R v.4.3.1. To generate the figures, the following R packages were used: tidyverse (Wickham *et al*., 2019), khroma (Frerebeau, 2023), ggrepel https://github.com/slowkow/ggrepel, readxl https://github.com/tidyverse/readxl, metbrewer (https://github.com/BlakeRMills/MetBrewer), and momacolors (https://github.com/BlakeRMills/MoMAColors).

### Plant materials

Germplasm and/or leaf material of *O. officinalis* (accessions W0002, W0065, W1131, W1313, and W1361) and *O. coarctata* (accession W0551), and genome DNA solutions of several accession of *O. officinalis* (W0002, W0566, 0614, W1131, W1200 and W1252) were provided by the Resource Bank of the National Institute of Genetics, Japan (http://www.shigen.nig.ac.jp/rice/oryzabase/locale/change).

### Momilactone measurements

Each harvested leaf blade was treated with 0.5 mM CuCl_2_ for 72 h before the analysis. The treated sample (roughly 100–500 mg) was submerged in 4 ml of 70% methanol at 4°C for 24 h.

For *O. officinalis*, 2 μl of the extract was subjected to LC‐MS/MS analysis (Sciex QTRAP3200) with the following selected reaction monitoring transitions used as previously described (Miyamoto *et al*., 2016): momilactone A, *m/z* 315/271; momilactone B, *m*/*z* 331/269.

For *O. coarctata*, plant material (100–150 mg) was weighed in a 2 ml extraction tube (CKMix, Bertin Technologies, Montigny le Bretonneux, France), and the tube filled up with methanol/water (70/30, v/v, 2 ml). After extractive grinding (3 × 30 s with 30 s breaks, 6000 rpm) using the bead beater (Precellys Homogenizer; Bertin Technologies, Montigny le Bretonneux, France), samples were incubated for 24 h at 4°C. After centrifugation, the supernatant was evaporated to dryness, resumed in acetonitrile (50 μl), and injected into the UHPLC–MS/MS system (5 μl).

A QTRAP 6500+ mass spectrometer (Sciex, Darmstadt, Germany) was used to acquire mass spectra and product ion spectra in positive electrospray ionization (ESI) mode. The MS/MS system was operated in the scheduled multiple reaction monitoring (MRM) mode at an ion spray voltage of 5500 V with the following ion source parameters: curtain gas (35 psi), temperature (550°C), gas 1 (55 psi), gas 2 (65 psi), and entrance potential (10 V). For analysis of momilactones A and B, the MS/MS parameters were tuned to achieve fragmentation of the [M + H]^+^ molecular ions into specific product ions: [M + H]^+^: momilactone A *m*/*z* 315.2/271.2 (quantifier) and *m*/*z* 315.2/269.2 (qualifier), momilactone B *m*/*z* 331.3/269.2 (quantifier) and *m*/*z* 331.2/128.1 (qualifier). For tuning, acetonitrile/water solutions of each analyte were introduced by means of flow injection using a syringe pump. The samples were separated by means of an ExionLC UHPLC (Shimadzu Europa GmbH, Duisburg, Germany) consisting of two LC pump systems ExionLC AD, an ExionLC degasser, an ExionLC AD autosampler, an ExionLC AC column oven and an ExionLC controller, and equipped with a BEH Premier C18 column (150 × 2.1 mm, 100 Å, 1.7 μm; Waters, Manchester, UK). Operated with a flow rate of 0.5 ml min^−1^ at a column oven temperature of 45°C using 0.1% formic acid in water (v/v) as solvent A and 0.1% formic acid in acetonitrile (v/v) as solvent B. Chromatography was performed with the following gradient: 70% B increased to 77.5% in 2.5 min, increased to 100% B in 0.5 min, held 1 min at 100% B, decreased to 70% B in 0.5 min, and held 0.5 min at 70% B. Data acquisition, and instrumental control was performed with Analyst 1.6.3 software (Sciex, Darmstadt, Germany).

### cDNA cloning of OoKSL4 and OcKSL4 and their functional analysis by metabolic engineering systems

The nucleotide sequences of full‐length *OoKSL4‐1*, *OoKSL4‐2* and *OcKSL4* have been deposited in the GenBank database with accession nos. LC831403, LC831404 and LC831406, respectively.

For *OoCPS4*, full length of cDNA was amplified by reverse transcription polymerase chain reaction (RT‐PCR), using cDNA prepared from RNA sample of *O. officinalis* leaf 24‐h after the CuCl_2_ treatment with forward primer (OoCPS4F: 5′‐ TTCTGCCCAAATTCGATGCCGGCCTTTACTGCATC‐3′), reverse primer (OoCPS4R: 5′‐ GTGATGGTGATGCCCAATCACATCTTGGAATATGA‐3′). Nucleotide sequence of OoCPS4 has been deposited in the GenBank database with accession no. LC831405. For *OoKSL4‐1* full‐length cDNA were amplified by RT‐PCR using forward primer (OoKSL4‐1F: 5′‐ TTCTGCCCAAATTCGATGGTTATCACCCATATTTTGAG‐3′), reverse primer (OoKSL4‐1R: 5′‐ GTGATGGTGATGCCCAGATGAACTTGTAGCACCTAG‐3′). To recover the nonsense G‐to‐A polymorphism in *OoKSL4‐2*, a 2‐step overlap PCR was performed. For the first amplicon, forward primer‐1 (OoKSL4‐2F1: 5′‐TTCTGCCCAAATTCGATGGCGAATCCTATGG‐3′) and reverse primer‐1 (OoKSL4‐2R1: 5′‐GCTACCTGACCAACAACCCATTT‐3′) were used. For the second amplicon, forward primer‐2 (OoKSL4‐2F2: 5′‐AAATGGGTTGTTGGTCAGGTAGC‐3′) and reverse primer‐2 (OoKSL4‐2R2: 5′‐GTGATGGTGATGCCCGTAGCCTATAGTTTTCAG‐3′) were used. The full length of the recovered *OoKSL4‐2* was then amplified using forward primer‐1 and reverse primer‐2 as the primer set, with amplicons from part 1 and part 2 serving as templates. For *OsDXS3* full‐length cDNA were amplified by RT‐PCR using forward primer (DXS3F: 5′‐ TTCTGCCCAAATTCGATGGCGCTCCAGGCATCG‐3′), reverse primer (DXS3R: 5′‐ GATGCATACCGGTCGTCAGCTGAGCTGAAGTGC‐3′). For *OsGGPPS* full‐length cDNA were amplified by RT‐PCR using forward primer (GGPSF: 5′‐ TTCTGCCCAAATTCGATGGCTGCCTTCCCCCCG‐3′), reverse primer (GGPSR: 5′‐ GATGCATACCGGTCGTCAGTTCTGCCGATAGGC‐3′).

Full‐length cDNAs of *OoCPS4*, *OoKSL4‐1* and *OoKSL4‐2*, containing transit peptides, and two genes from *O. sativa* encoding OsDXS3 and OsGGPPS in the MEP pathway were, respectively, cloned into NruI‐SmaI sites of pEAQ‐HT vector by In‐Fusion cloning system (Takarabio‐Clontech, Kusatsu, Shiga, Japan). Constructed pEAQ‐HT‐*OoffKSL4‐1*, pEAQ‐HT‐*OoffKSL4‐2*, pEAQ‐HT‐*OsDXS3* and pEAQ‐HT‐*OsGGPPS*, all with 6× His tags at the C‐terminal region, were then transferred into *Agrobacterium tumefaciens* for agroinfiltration.

In the *Nicotiana benthamiana* transient expression system, 5‐wk‐old plants grown in a plant incubator (23°C, 16 h : 8 h, light : dark photoperiod) were used. After agroinfiltration, the plants were further grown for 5 d under the same conditions. The leaves were then harvested, cut into 5 mm squares, immersed in *n*‐hexane for a one‐day extraction. Extraction and purification were done as described previously (Toyomasu *et al*., 2018).

The cDNA encoding partial OoKSL4‐1 for functional analyses using *Escherichia coli* was amplified by RT‐PCR using primers 5′‐GGATCCATGGCGAATCCTGTGGAAG‐3′ (forward; BamHI site is underlined) and 5′‐GTCGACTTAAGATGAACTTGTAGCACCTAG‐3′ (reverse; SalI site is underlined), and subcloned into pGEX‐4T‐3 (GE Healthcare) using BamHI and SalI. The cDNA encoding pseudo‐mature OoKSL4‐2 was amplified by RT‐PCR using forward primer 5′‐GGTTCCGCGTGGATCCGAGGCTAGAATAAGGAGGC‐3′ and reverse primer 5′‐GGGAATTCGGGGATCCCTAGTAGCCTATAGTTTTC‐3′ (terminal sequences of pGEX‐4T‐3 digested by BamHI are underlined), and inserted into BamHI‐digested pGEX‐4T‐3 by using In‐Fusion Smart Assembly Cloning Kit (Takarabio‐Clontech). Full‐length *OcKSL4* cDNA was amplified by RT‐PCR using primers 5′‐GGATCCATGGCGAATTATCCCATGGAG‐3′ (forward; BamHI site is underlined) and 5′‐TTAAGATGGACTAGTAGCCTCTAGTTTAAGTGGC‐3′ (reverse; NotI site is underlined), and subcloned into pGEM‐T‐Easy. *OcKSL4* cDNA was inserted into pGEX‐4T‐3 using BamHI and NotI. For RT‐PCR, we used cDNA prepared from RNA from leaf blades of *O. officinalis* W0002 and *O. coarctata* W0551 24 h after UV treatment. OoKSL4‐2_445W was generated by mutagenesis PCR using OoKSL4‐2 in pGEX as template, with primers 5′‐gTCAGGTAGCTTATTGAAAC‐3′ (forward) and 5′‐caACAACCCATTTTCTCTAGGATTG‐3′ (reverse; small characters indicate W codon for mutagenesis).

The functional analyses of OoKSL4‐1, OoKSL4‐2 and OcKSL4 in a metabolic engineering system using *E. coli* cells were performed as described previously (Toyomasu *et al*., 2016). The *n*‐hexane extract from culture medium containing recombinant *E. coli* BL21‐DE3 harbouring pRSF‐PaGGS:OrCPS4 and a respective construct to express one of the above proteins was subjected to GC‐MS analysis. PaGGS is a geranylgeranyl synthase domain of fusicoccadiene synthase in *Phomopsis amygdali* (PaFS) (Toyomasu *et al*., 2007) and OrCPS4 is a *syn*‐CDP synthase from *O. rufipogon* W0106 (Miyamoto *et al*., 2016), to supply GGDP and *syn*‐CDP, respectively.

GC‐MS was conducted using an Agilent 8860 GC‐5977 MSD system, fitted with a fused silica, chemically bonded capillary column (HP‐1MS; 0.25 mm in diameter, 60 m long, 0.25 μm film thickness). Ionization voltage was 70 eV. Each sample was injected onto the column at 250°C in the splitless mode. After a 2‐min isothermal hold at 60°C, the column temperature was increased by 30°C min^−1^ to 150°C, 10°C min^−1^ to 180°C, 2°C min^−1^ to 210°C, and 30°C min^−1^ to 300°C. The flow rate of the helium carrier gas was 1 ml min^−1^. We used 9βH‐pimara‐7,15‐diene as a standard, which was synthesized as previously described (Ye *et al*., 2017).

### Semi‐quantitative RT‐PCR

*Oryza officinalis* leaf tissue from six genotypes was collected after 72 h of treatment with either water or 500 μM CuCl₂. RNA was extracted using the RNeasy Plant Mini Kit (Qiagen) following the manufacturer's protocol. Reverse transcription and cDNA synthesis was performed using the PrimeScript™ RT Reagent Kit with gDNA Eraser (TaKaRa, Kusatsu, Shiga, Japan).

The PCR was carried out using Ex Premier™ DNA Polymerase Dye Plus (TaKaRa). After amplification, an equal volume of the reaction mixture was loaded onto a 1% agarose gel, followed by gel electrophoresis for 40 min. For *OoKSL4‐1* and *OoKSL4‐2*, the same primers were used as for the cloning of the CDS (as mentioned in the previous section). For amplification of the *OoUBQ* reference gene, we used forward primer 5′‐CATGCTTAGTGGGGTTCGTG‐3′ and reverse primer 5′‐GAGCTGAGTGAGCTGTGTTG‐3′.

## Results

### The momilactone biosynthetic gene cluster is prevalent throughout the *Oryza* genus

Among the 11 different genome types within the genus *Oryza*, the MBGC has been extensively studied in only 3 lineages. The cluster exhibits a high level of conservation among *Oryza* species of the AA and BB genome groups, whereas it is incomplete in the FF species *O. brachyantha* (Fig. 1b) (Miyamoto *et al*., 2016). To better understand the evolution of MBGC in the different *Oryza* lineages, we mined newly published genome assemblies (Table S1) for the presence and location of the MBGC (see the Materials and Methods section for details). Our analysis included genomes of the diploid species *O. officinalis*, *O. eichingeri*, *O. rhizomatis* (all CC), *O. australiensis* (EE), and *O. granulata* (GG), as well as of the allotetraploid species *O. alta* (CCDD) and *O. coarctata* (KKLL). We also included *Leersia perrieri* as an outgroup to *Oryza* in our analyses. We used OrthoFinder (Emms & Kelly, 2019) to identify orthogroups containing putative *O. sativa* momilactone biosynthetic genes (Fig. 1a). Based on phylogenetic relationships between the set of genes in each orthogroup, we considered as homologs those genes that belonged to the same clade as the *O. sativa* momilactone biosynthetic genes (Figs S2–S5). We further mined the genomic regions upstream and downstream of the identified homologs to confirm their organization in a genomic cluster.

We detected the MBGC in the CC genome species *O. officinalis*, and the corresponding genes across different scaffolds of two other CC species, *O. eichingeri*, and *O. rhizomatis*. Within the CC lineage, gene copy numbers varied, and for some genes we found signatures of species‐dependent duplications. In *O. officinalis* (CC), the cluster (*c*. 385 kb in length) was located on chromosome 4 and contained *CPS4*, *MAS*, two copies of *KSL4*, and two *CYP99A2/3* orthologues (Fig. 1b). In *O. eichingeri* and *O. rhizomatis* (both CC), we identified orthologues of *CPS4*, *MAS*, two copies of *KSL4*, and *CYP99A2/3*; *O. eichingeri* carried two copies each for *CYP99A2/3* and *MAS*, with the second copy of the latter appearing pseudogenic (Fig. 1b). In *O. rhizomatis*, the *CYP99A2/3* and one of the *KSL4* paralogs co‐occurred on Scaffold2280, *CPS4* and the other *KSL4* paralog on Scaffold1491 (Fig. 1b). Since the respective genome assemblies are fragmented, scaffold‐level assemblies, it remains speculative whether the genes in these two species are organised in an actual biosynthetic gene cluster. In agreement with the presence of the cluster in the CC species *O. officinalis* and, presumably, in *O. eichingeri* and *O. rhizomatis*, we identified a *c*. 520 kb MBGC cluster on chromosome 4 of sub‐genome C of the allotetraploid *O. alta* (CCDD). This cluster contained orthologues of *MAS*, *KSL4*, *CYP99A2/3*, and three orthologues of *CPS4* (Fig. 1b). By contrast, we did not find any orthologues of momilactone biosynthetic genes in the D sub‐genome of *O. alta*. This observation is in line with *O. australiensis* (EE), the closest relative of the *O. alta* D‐type sub‐genome lacking a MBGC (the ancestral diploid D‐type donor is presumably extinct) (Ge *et al*., 1999; Bao & Ge, 2004). In the allotetraploid *O. coarctata* (KKLL), we identified a MBGC spanning *c* . 55 kb and containing one orthologue each of *CPS4*, *MAS*, *KSL4*, and *CYP99A2/3*. Based on a species tree obtained from 4069 multiple‐sequence alignments of single‐copy orthologues (Fig. 1b) and from existing literature (Ge *et al*., 1999; Guo & Ge, 2005; Shenton *et al*., 2020), we could tentatively assign the MBGC in *O. coarctata* to chromosome 4 (LG08) of the LL sub‐genome (evenly numbered chromosomes in the annotation belong to the LL, unevenly numbered ones to the KK sub‐genome). Because this lineage diverged earlier than the MBGC‐lacking EE lineage (Fig. 1b), we speculate that the cluster most likely became lost in the EE lineage. Lastly, we did not detect the cluster or homologs thereof in *O. granulata* (GG).

To further confirm that we had detected the MBGC and not another diterpene‐related BGC, we analysed the synteny of the corresponding regions among the different species. We hypothesized that if the MBGC in the *Oryza* genus had a common origin, one should observe high synteny of the genomic region among all species. We excluded *O. rhizomatis* and *O. eichingeri* from the synteny analysis, because in those genome assemblies the momilactone biosynthesis genes were located on short scaffolds that often carried only very few (*c*. 3–4) genes in total. For the remaining species and sub‐genomes, the genomic region containing the MBGC (or lacking it, as in the case of *O. alta* sub‐genome D, *O. australiensis*, *O. coarctata* sub‐genome K, and *O. granulata*) was syntenic (Figs 1c, S6). The synteny further confirmed the presence of the MBGC in the different reference genomes and indicated that this region shares a common evolutionary origin. These results suggest that the MBGC is prevalent at a basal position of the *Oryza* phylogeny. The evolution of the cluster in different lineages is characterized by differences in cluster size, lineage‐specific rearrangements with an important variation in gene copy number, and lineage‐specific loss of the cluster.

### Momilactone production in different *Oryza* lineages

Among the investigated *Oryza* genome types (CC, CCDD, EE, KKLL and GG), only species and sub‐genomes belonging to the lineages CC and LL include an orthologous MBGC. To assess the functionality of the predicted clusters in the CC and LL lineages, we proceeded to evaluate the momilactone‐producing potential of their respective representative species *O. officinalis* (CC) and *O. coarctata* (KKLL).

**Fig. 2:**
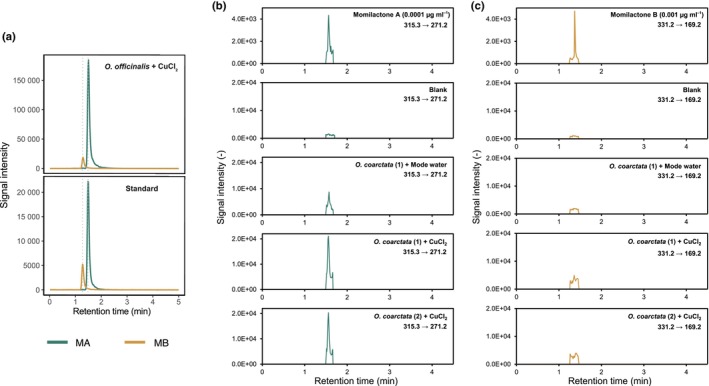
Analysis of momilactones in representative species of the *Oryza* genus. (a) Extracts from *Oryza officinalis* CuCl_2_‐treated leaf blades were analysed by liquid chromatography–tandem mass spectrometry (LC‐MS/MS). Five μl of the extract were subjected to LC‐MS/MS under the conditions described in the Materials and Methods section. Momilactones were detected with combinations of *m/z* 315/271 for momilactone A and *m*/*z* 331/269 for momilactone B in the multiple reaction‐monitoring mode. MA, momilactone A; MB, momilactone. (b, c) Ultrahigh performance liquid chromatography (UHPLC)‐MS/MS (ESI^+^) chromatograms in the scheduled MRM mode of momilactone A (b) and momilactone B (c) in reference solution (0.0001 μg ml^−1^), mode water *Oryza coarctata*, CuCl_2_‐treated leaf blades of *O. coarctata* replicate 1, and CuCl_2_‐treated leaf blades of *O. coarctata* replicate 2 (from upper panel to lower panel), showing the specific mass transition *m*/*z* 315.3/271.2 and *m*/*z* 331.2/169.2 used for quantitation of momilactone A and B, respectively.

We conducted liquid chromatography–tandem mass spectrometry (LC‐MS/MS) or ultra‐high‐performance liquid chromatography (UHPLC)‐MS/MS to detect momilactones A and B in leaf blades. Momilactone basal levels can be close to detection limits, which is why we induced momilactone production by treating the plants with CuCl_2_ 3 d before extraction, a treatment previously shown to increase momilactone levels in different *Oryza* species (Miyamoto *et al*., 2016). Consistent with the presence of the MBGC and the similar expression pattern of the corresponding genes upon induction (Fig. S1), we detected both momilactone A and B in several *O. officinalis* lineages (Figs 2a, S7), but only momilactone A in *O. coarctata* (Fig. 2b,c). Even after induction with CuCl_2_, *O. coarctata* accumulated only low levels of momilactone A (Fig. 2b). It should be noted that momilactone B tends to be the minor constituent, hence absence of momilactone B in *O. coarctata* could also be due to limitations in detection range. Taken together, these results provide evidence for the functionality of the identified MBGC in the CC and KKLL species *O. officinalis* and *O. coarctata*, respectively.

### Functional analysis of KSL4 in *O. officinalis* and *O. coarctata*

**Fig. 3:**
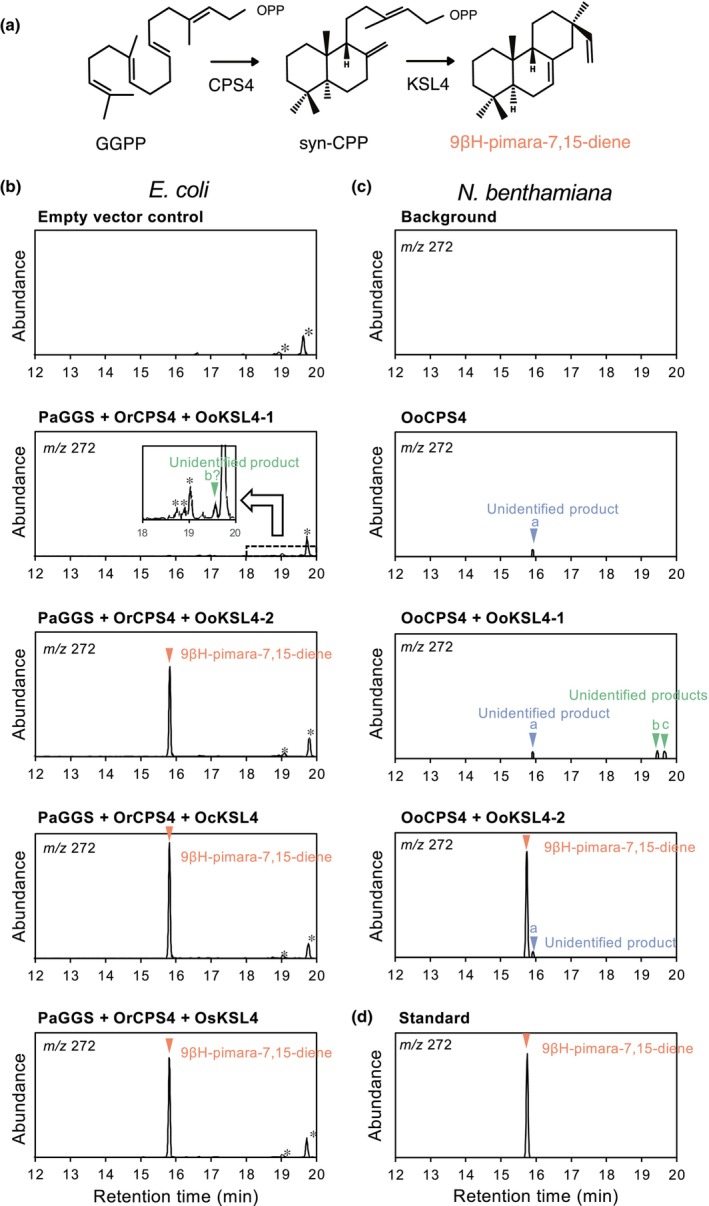
Functional characterization of KSL4 orthologues. (a) Simplified representation of the momilactone biosynthetic pathway steps catalysed by CPS4 and KSL4. (b) GC‐MS chromatograms of the products obtained from recombinant OoKSL4‐1, OoKSL4‐2 (with the reverted Trp at position 445), OcKSL4, and OsKSL4 from *Escherichia coli* in a metabolic engineering system. PaGGS: GGDP Synthase from *Phomopsis amygdali*; OrCPS4: CPS4 from *Oryza rufipogon* (c) GC‐MS chromatograms of the products obtained in the *Nicotiana benthamiana* transient expression system after expressing *OoCPS4*, *OoCPS4* + *OoKSL4‐1* and *OoCPS4* + *OoKSL4‐2*. (d) GC‐MS chromatograms of the 9βH‐pimara‐7,15‐diene standard. Orange triangles indicate production of 9βH‐pimara‐7,15‐diene. Nonspecific peaks from *E. coli* cells are indicated with asterisks (*). Mass spectra are included in Supporting Information Fig. S10.

After establishing the existence of orthologous MBGCs in wild relatives of cultivated rice from the *Oryza* genus and confirming the presence of momilactone A and B in *O. officinalis* and momilactone A in *O. coarctata*, we next wanted to confirm the enzymatic functionality of the orthologues from those clusters. We focused on KSL4, which is known to catalyse the first dedicated step towards momilactone biosynthesis (i.e. the conversion of *syn*‐CDP to 9βH‐pimara‐7,15‐diene) (Fig. 3a). We tested the enzymatic activity of the KSL4 orthologues in representative species of the late‐diverged CC and early‐diverged KKLL lineages: OoKSL4‐1 and OoKSL4‐2 from *O. officinalis*, and OcKSL4 from *O. coarctata*.

The available annotation of the genes in the MBGC in *O. officinalis* showed inconsistencies, such as an extra sequence of amino acids in the C‐terminal part of OoKSL4‐1 and a missing exon in both *OoKSL4‐1* and *OoKSL4‐2*, compared to the orthologue in *O. sativa* (*OsKSL4*) (Fig. S8). These discrepancies may be attributed to limitations in the genome annotation process, which relied on RNA‐Seq data from tissues in which the constitutive expression of momilactone biosynthetic genes is generally low or absent (Fig. S1) (Shenton *et al*., 2020).

Exposure to UV light is known to induce the accumulation of momilactones in rice leaves (Kodama *et al*., 1988). To accurately investigate the function of KSL4 in *O. officinalis* and *O. coarctata*, we used previously published mRNA‐Seq data from UV‐irradiated leaf sheaths of both species to assemble a *de novo* transcriptome (Itoh *et al*., 2021). Subsequently, we conducted a homology search for OsKSL4 homologs in *O. officinalis* and *O. coarctata*. Using the assembled contig sequences as a reference, we designed specific primers and successfully cloned the cDNAs of these three *KSL4* homologs. In contradiction to the *O. officinalis* W0002 reference genome sequence (Shenton *et al*., 2020), our OoKSL4‐2 cDNA clones showed a premature stop codon at amino acid position 445 (Figs S8, S9). By investigating 14 additional accessions, we determined that this mutation was private to the W0002 line distributed by the stock centre and hence is not representative of the consensus sequence at the population level (Fig. S9). This may also explain why we detected much lower momilactone levels in the reference strain W0002 compared to other genotypes (Fig. S7). We therefore reverted the G‐to‐A polymorphism in our cDNA clone to the consensus reference sequence of *OoKSL4‐2*. To confirm the enzymatic activity of OoKSL4‐1, OoKSL4‐2 and OcKSL4 in *E. coli*, we co‐expressed each one together with recombinant GGPP Synthase (GGS) from *Phomopsis amygdali* and CPS4 from *O. rufipogon*, which provide the substrate *syn*‐CDP (Fig. 1a; Toyomasu *et al*., 2007, 2018), and analysed the products using gas chromatography coupled to mass spectrometry (GC‐MS). The retention time and mass spectra of the product generated by OoKSL4‐2 and OcKSL4 were identical to those of 9βH‐pimara‐7,15‐diene, indicating that both enzymes have the same enzymatic activity as OsKSL4 (Figs 3b, S10). By contrast, we did not observe any production of 9βH‐pimara‐7,15‐diene by OoKSL4‐1, but production of minute amounts of an unidentified product (labelled as ‘b’ in Fig. 3b). We were able to corroborate the production of 9βH‐pimara‐7,15‐diene by OoKSL4‐2 by transiently expressing OoKSL4‐1 and OoKSL4‐2 together with OoCPS4 in *Agrobacterium tumefaciens*‐infiltrated *Nicotiana benthamiana* leaves (Figs 3c, S10). We therefore conclude that OoKSL4‐2 is the enzyme responsible for catalysing the conversion of *syn*‐CDP to 9βH‐pimara‐7,15‐diene in *O. officinalis*, which was further supported by *OoKSL4‐2* transcript levels being more strongly induced by CuCl_2_ than those of *OoKSL4‐1* in all tested *O. officinalis* accessions except in the mutation‐carrying W0002 (Fig. S7).

### Species‐specific deviations in the MBGC architecture in the *Oryza* genus

**Fig. 4:**
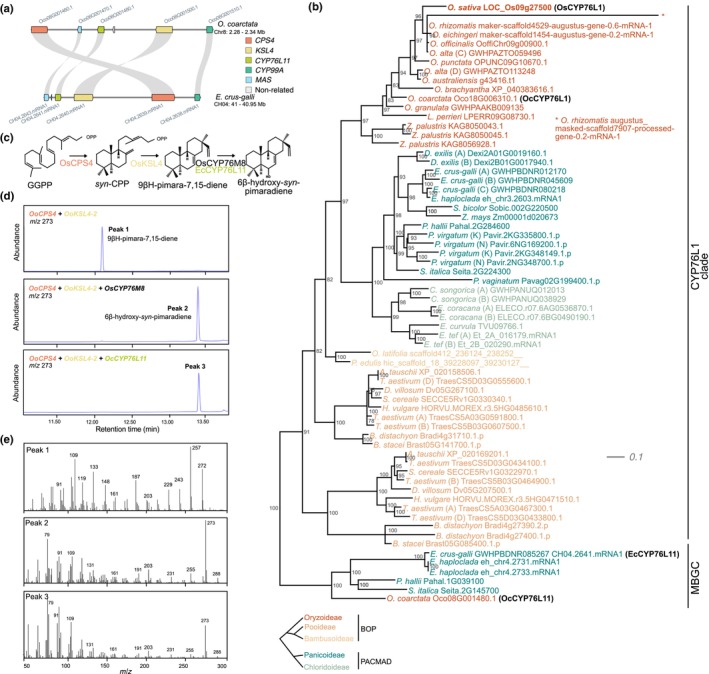
Phylogenetic and functional analysis of a newly identified CYP orthologue in the MBGC. (a) Microsynteny between MBGC in *Oryza coarctata* and *Echinochloa crus‐galli*. Note that this microsynteny does not extend to the flanking regions of the cluster (also see Supporting Information Fig. S11). Grey lines connect the respective orthologues (b) Subtree extracted from the maximum‐likelihood tree with the best‐fit model selected by ModelFinder representing the relationship between the orthogroup containing *O. coarctata* CYP76L11 (see also Figs S14, S15). The phylogenetic tree is based on amino acid sequence alignments of the OsCYP76L1 clade, EcCYP76L11 and OcCYP76L11 (both present in the *E. crus‐galli* and *O. coarctata* MBGC, respectively), and *Poaceae* species belonging to the subfamilies Oryzoideae, Pooideae, Bambusoideae, Panicoideae and Chloridoideae. The phylogenetic relation between these subfamilies is represented in the bottom graph. (c) Simplified representation of the momilactone biosynthetic pathway steps, indicating the functional equivalence of CYP76M8 and CYP76L11. (d) GC‐MS chromatograms of the extracts from *Nicotiana benthamiana* leaves following agroinfiltration with *OoCPS4* + *OoKSL4‐2*, *OoCPS4* + *OoKSL4‐2* + *OsCYP76M8*, *OoCPS4* + *OoKSL4‐2* + *OcCYP76L11*. OsCYP76M8 has previously been reported to yield 6β‐hydroxy‐*syn*‐pimaradiene (peak 2) in *in vitro* assays (Wang *et al*., 2012). (e) Mass spectra of products from three groups scanned in selected ion mode (*m*/*z* = 273). The extracts were subjected to GC‐MS analysis using a Shimadzu GC‐MS‐QP2020 instrument with the following parameters: 70 eV, electron ionization, positive ion mode; SH‐Rxi‐5MS column using a temperature program of *c*. 70–280°C and a temperature gradient of 15°C min^−1^.

We next investigated whether there were any noticeable species‐specific deviations in the MBGC architecture. In *O. coarctata*, we identified an extra gene located between *MAS* and *KSL4* and encoding a CYP. Importantly, this enzyme does not belong to the CYP99A subfamily, which is the canonical CYP in the MBGC of the other *Oryza* species (Fig. 1b). Instead, the MBGC in *O. coarctata* strikingly resembled that of *E. crus‐galli* (Fig. 4a), which also contains an additional CYP‐encoding gene, *EcCYP76L11*, that does not belong to the CYP99A subfamily either (Guo *et al*., 2017; Kitaoka *et al*., 2021). EcCYP76L11 catalyses the same reaction as OsCYP76M8, converting 9βH‐pimara‐7,15‐diene to 6β‐hydroxy‐*syn*‐pimaradiene (Kitaoka *et al*., 2021; Wang *et al*., 2012). In *O. sativa*, *OsCYP76M8* is located outside of the MBGC, which suggests that *E. crus‐galli* possibly contains a more compacted version of the cluster (Kitaoka *et al*., 2021).

We further investigated the MBGC in *O. coarctata*. An initial microsynteny comparison of the MBGCs in *O. coarctata* and *E. crus‐galli* revealed that the additional CYP‐encoding gene could indeed be an orthologue of *EcCYP76L11* (Fig. 4a). Notably, the microsynteny pattern was restricted to the MBGC itself and not to the flanking regions of the cluster in both species (Fig. S11). We confirmed that this additional CYP‐encoding gene was a reciprocal best hit to EcCYP76L11. To verify the relationship between the CYP‐encoding gene in *O. coarctata* MBGC and *EcCYP76L11*, we conducted an amino acid sequence‐based phylogenetic analysis including all *Oryza* species from our panel and different representative species of the main Poaceae subfamilies (Fig. S12; see the Materials and Methods section for details). Our analysis placed the *O. coarctata* CYP (Oc08G001480) in a clade with EcCYP76L11 (CH04.2641), the two CYP76L11 paralogs from the MBGC of *E. haploclada* (eh_chr4.2731 and eh_chr4.2733) (Wu *et al*., 2022a,b), and two CYPs from *P. hallii* (Pahal.1G039100) and *S. italica* (Seita.2G145700), respectively (Fig. 4b). Interestingly, the *O. coarctata* CYPs did not group with the only CYP76L member from *O. sativa* (OsCYP76L1) or the CYP76L1 orthologues in other *Oryza* species (including Oc18G006310 from *O. coarctata*) (Fig. 4b). Consequently, based on the similarity to EcCYP76L11, we named the newly identified *O. coarctata* CYP‐encoding gene *OcCYP76L11*. In *E. crus‐galli*, EcCYP76L11 converts 9βH‐pimara‐7,15‐diene to 6β‐hydroxy‐*syn*‐pimaradiene (Fig. 4c; Kitaoka *et al*., 2021), analogously to CYP76M8 in *O. sativa*. When transiently co‐expressing OcCYP76L11 together with OoCPS4 and OoKSL4 in *N. benthamiana* leaves, we were able to detect 6β‐hydroxy‐*syn*‐pimaradiene (Fig. 4d,e), underlining the functional homology between EcCYP76L11 and OcCYP76L11. Our analysis suggests that *OcCYP76L11* was present in the ancestral *Oryza* MBGC and was retained in the early‐divergent *O. coarctata*, while it became lost in the later‐diverging clades of the *Oryza* genus. Taken together, these results further support that the orthologous MBGC that we identified in *O. officinalis* and *O. coarctata* are functional and responsible for the production of momilactones. Notably, *O. coarctata* (KKLL), the earliest‐divergent *Oryza* species with a MBGC, showed a distinct cluster architecture.

## Discussion

### The momilactone BGC shows diversified architecture within the *Oryza* genus

BGCs arise from the duplication of enzymes involved in primary metabolism, followed by neofunctionalization and/or relocation processes that are influenced by positive‐ and negative‐selection pressures (Smit & Lichman, 2022; Polturak *et al*., 2022b). Ecological factors shape the direction and strength of the selection process, determining the evolution of the metabolism and ultimately affecting the architecture of BGCs. Here, we studied the composition and evolution of the momilactone biosynthetic gene cluster in previously unexplored lineages in the genus *Oryza*. We provide evidence that the MBGC is prevalent in the CC and LL lineages, while it is absent in the intermediate EE or the basal GG lineages (Fig. 1b). Our data show that the evolution of the MBGC is marked by lineage‐specific rearrangements, such as gene copy number variation or the increase of the size of the cluster, on‐going pseudogenization in certain paralogs, and occasional cluster loss. Moreover, we found a distinct MBGC architecture in *O. coarctata*, with the cluster in that species harbouring a close orthologue of *EcCYP76L11*. This finding represents the first instance of an alternative architecture of the MBGC in *Oryza* and strengthens the idea of a common origin of the MBGC in *Echinochloa* and *Oryza*. Overall, our research sheds light on the evolutionary dynamics and distribution of the MBGC in the genus *Oryza*.

In our study, we found the MBGC at a basal position of the *Oryza* phylogeny in the species *O. coarctata* (sub‐genome LL). *Oryza coarctata* is a polyploid species resulting from the hybridization of the KK and LL diploid genomes; the respective ancestral species have presumably gone extinct or remain to be discovered (Ge *et al*., 1999; Lu *et al*., 2009). The KK sub‐genome occupies a basal position relative to the EE and DD lineages, while the *O. coarctata* LL sub‐genome (initially designated as HH) occupies a more basal position compared to KK and it is closer to the diploid FF genome species *O. brachyantha*. Since we found the cluster in *O. coarctata*, a species that branched off before the divergence of the EE lineage, we hypothesize that *O. australiensis* (EE) likely lost the MBGC. Our study thereby is one of the very few to document a case of inter‐specific loss of biosynthetic gene clusters (Smit & Lichman, 2022). A lineage‐specific loss of a BGC was previously described in *Oryza* for the phytocassane biosynthetic gene cluster: *Oryza* AA species and the outgroup species *L. perrieri* and *Zizania palustris* possess the phytocassane cluster, whereas it was partially lost in *O. punctata* (BB) and *O. brachyantha* (FF) (Miyamoto *et al*., 2016; Yan *et al*., 2022). It is worth noting that the MBGC is also found in the *Panicoideae* and *Chloridoideae* subfamilies of the *Poaceae* and most likely evolved from a common ancestor (Wu *et al*., 2022a). However, the distribution of the cluster in the PACMAD clade is restricted to certain tribes, while it is either fragmented or completely lost in others (Wu *et al*., 2022a). In line with these observations, our results highlight the dynamic nature of biosynthetic gene clusters and suggest that loss of BGCs might be prevalent within *Poaceae*.

The MBGC and MBGC‐like clusters are widespread in the *Poaceae* family (Wu *et al*., 2022a). Species belonging to the *Triticeae* tribe (Pooideae), phylogenetically more closely related to *Oryza* than to *Echinochloa*, possess a functionally distinct diterpene cluster that shares similarities with the MBGC (Polturak *et al*., 2022a; Wu *et al*., 2022b; Liu *et al*., 2024). This MBGC‐like is located on the homoeologous chromosomes 2A and 2D in wheat (*Triticum aestivum*), homologs of rice chromosome 4, and exhibits synteny with the region containing the MBGC in rice (Polturak *et al*., 2022a). However, the cluster in *Triticeae* lacks certain important momilactone biosynthesis genes, for example a *MAS* and a *syn‐CPS* to produce *syn* labdane‐related diterpenoids. Instead, the *Triticeae* cluster produces normal labdane‐related diterpenoids in wheat (*Triticum aestivum*) and barley (*Hordeum vulgare*) (Polturak *et al*., 2022a; Liu *et al*., 2024). Owing to the shared synteny between the MBGC in rice and the MBGC‐like in wheat, Polturak *et al*. (2022a) suggested that the *Triticeae* diterpene cluster and the MBGC share a common evolutionary origin, and hypothesized that both clusters could have originated from a common ancestral BGC before the divergence of the PACMAD and BOP clades. Wu *et al*. (2022a), who performed a more extensive survey of the MBGC in different Poaceae genomes, alternatively attributed the evolution of the cluster in Poaceae to multiple instances of lateral gene transfer (LGT) and proposed that the MBGCs found in the PACMAD clade (Panicoideae and Chloridoideae subfamilies) originated from the MBGC‐like cluster in *Triticeae*. They moreover speculated that *EcCYP76L11* in *Echinochloa* was also laterally transferred from the *Triticeae* clade along with the MBGC‐like. After the PACMAD species acquired the MBGC‐like, it would then have recruited *MAS*. Subsequently, another LGT event would have had to occur, resulting in the acquisition of the cluster by *Oryzoideae*, followed by the loss of the *CYP76L11* gene (Wu *et al*., 2022a). Wu *et al*. based their conclusions on the phylogenetic incongruences among the MBGC orthologous genes, topology tests on the constrained trees for each MBGC gene, and a general lack of synteny between the genomic region containing the MBGC in the different tribes in the *Poaceae*. It is worth noting that the MBGC in *Panicoideae* and *Chloridoideae* is also not syntenic, following the same reasoning, this would imply that these gene clusters were acquired through two separate and independent lateral gene transfer (LGT) events rather than evolving from a common ancestor, as suggested by the same authors. There are several phylogenomic studies that suggest that LGT is prevalent among grasses; LGTs in *Poaceae* usually occur among closely related species, while the insertion occurs in random and nonsyntenic genomic regions; however, the underlying mechanisms by which natural plant–plant LGT would occur are still largely unknown (Dunning *et al*., 2019; Hibdige *et al*., 2021). Our findings provide evidence that the *CYP76L11* gene has not been completely lost in the genus *Oryza*. The discovery of *CYP76L11* in *O. coarctata* implies either a common origin by vertical transmission of the MBGC between *Echinochloa* and *Oryza*, or a lateral gene transfer (LGT), as proposed by Wu *et al*. (2022a,b). However, the findings from both Polturak *et al*. (2022a) and our study (Fig. S13) indicate high synteny between the flanking regions containing the MBGC‐like cluster in *Triticeae* and the MBGC in *Oryza* (including *O. coarctata*) and thus suggest a common evolutionary origin of this genomic region, including the MBGC. This suggests that there may have been a common ancestor of the MBGC that existed before *Oryza* and *Triticeae* diverged. However, it is difficult to reconcile the synteny results shown by us and Polturak *et al*. (2022a) with the phylogenomic signatures of LGT shown by Wu *et al*., especially considering that the MBGC in *Echinochloa* and *Oryza* produces the same type of compounds, unlike in *Triticeae*. To shed light onto this question, it is important to consider the impact and prevalence of gene and MBGC losses in *Poaceae*. The presence or absence of MBGCs in different species can have significant implications for phylogenomic signals and evolutionary dynamics. The loss of MBGCs in certain lineages could potentially shape the phylogenomic patterns observed in Poaceae. Further investigations into the frequency and significance of MBGC losses across Poaceae will contribute to a better understanding of the evolutionary history of this cluster.

It is worth noting that *O. coarctata*, which possesses a CYP76L11 orthologue, is the only MGBC‐containing species lacking a CYP76M8 orthologue (Figs S14, S15). This suggests that the emergence of CYP76M8 from a duplication of a CYP76M7/8 ancestor after the branching of the KK lineage may have enabled the loss of CYP76L11.

### Allotetraploid rice species carry only one MBGC copy in their genome

In our study, we aimed to investigate whether allotetraploid species retain two copies of the MBGC. Previous research has shown that whole genome duplication (WGD) can lead to the silencing and progressive loss of one copy of a biosynthetic gene cluster, likely to balance the effects of increased gene dosage (Yang *et al*., 2021; Polturak *et al*., 2022a). We found that each of the two allotetraploid species we studied had retained one copy of the MBGC. In *Oryza alta* (CCDD), the cluster was present in the CC but not the DD sub‐genome, which means that the MBGC was either lost in the DD sub‐genome after hybridization or that the DD ancestor had already lost it before the hybridisation. Given that *O. australiensis* (EE), the closest relative of the *O. alta* D‐type sub‐genome (the ancestral diploid D‐type donor is presumably extinct), lacks a MBGC, it is possible that the cluster had already been lost in the DD donor.

Similarly, we identified only one copy of the MBGC in the *O. coarctata* LL but not the KK sub‐genome. The LL sub‐genome derives from a donor species that diverged earlier than the KK sub‐genome and is more closely related to *O. brachyantha* (FF), a species that harbours an incomplete MBGC (Guo & Ge, 2005; Miyamoto *et al*., 2016). However, we cannot say with certainty whether the cluster was lost in the KK sub‐genome after hybridization or was already lost before the hybridisation in the ancestor. Future studies using yet‐to‐be generated haplotype‐resolved assemblies of BBCC hybrid species could contribute to a better understanding of cluster evolution in allotetraploid rice, as both ancestors of these hybrids harbour the MBGC.

### Momilactone B formation may be a recent innovation in the *Oryza* genus

At the interspecies level, the existence of conserved BGCs does not always align consistently with the production of identical metabolites, while the metabolites derived from orthologous BGCs can also be species‐specific. The resulting divergence in chemotypes between species is associated with variations in biosynthetic genes external to the BGC, genes within the BGC, or transcription factors controlling the biosynthesis of these compounds (Zhou *et al*., 2016; Liu *et al*., 2020). In some cases, the function of the BGC is retained, and orthologous BGCs produce the same molecule. This is exemplified by *Oryza* species from the AA and BB lineages, which produce momilactones A and B (Miyamoto *et al*., 2016).

In our study, we show that the function of the MBGC is not only retained in the AA and BB but also the CC and LL lineages. Specifically, we found that the CC lineage, represented by *O. officinalis*, possesses an MBGC with slight differences in gene copy number, and that both momilactone A and B can be detected. The KKLL species *O. coarctata* contains an orthologous and syntenic MBGC with a distinct architecture compared to *O. sativa*. In line with our phylogenomic analysis suggesting the potential for momilactone production in *O. coarctata* and the co‐expression of its MBGC genes, we were able to detect momilactone A in leaf blades treated with CuCl_2_, which is known to induce momilactone production (Miyamoto *et al*., 2016). However, we were not able to detect momilactone B in *O. coarctata* within the limitations of our instrumental setup. Among the auxiliary genes outside the MBGC, *CYP701A8* and *CYP76M8* are necessary for the production of momilactone A, while CYP76M14 catalyses the conversion of momilactone A to momilactone B (Fig. 1a) (De La Peña & Sattely, 2021; Kitaoka *et al*., 2021). The fact that we were not able to detect momilactone B in *O. coarctata* could be attributed to the suppressed expression of the *CYP76M14* orthologue (*Oc01G012750*), which was only weakly expressed (< 3 TPM) upon UV treatment and barely detectable under control conditions in different tissues (Fig. S1). Intriguingly, unlike the MBGC genes, which are located on the LL sub‐genome in *O. coarctata*, the only copy of *OcCYP76M14* is located on the KK sub‐genome. To date, among the Poaceae, momilactone B has been detected only in AA, BB (Miyamoto *et al*., 2016) and CC *Oryza* species (this study). At a larger phylogenetic scale, *CYP76M14* is exclusive to *Oryza*, specifically to AA, BB, CC and KK lineages, and appears to have arisen from a duplication within the CYP76M subfamily (Fig. S14). It is imaginable that the production of momilactone B might have evolved after the divergence of the KK lineage by recruiting *CYP76M14* into the momilactone regulatory network.

## Competing interests

None declared.

## Author contributions

SP‐C, KO and CB conceived the study. SP‐C carried out the genomic and phylogenetic analyses. YL carried out CYP76L11 enzymatic and KSL4 expression assays. TT and YH carried out *in vitro* enzymatic assays on the functional analysis of KSL4. MG, YL and KO carried out momilactone measurements. HN, CD, KO and CB supervised the work. SP‐C and CB wrote the manuscript with contributions from all authors.

## Disclaimer

The New Phytologist Foundation remains neutral with regard to jurisdictional claims in maps and in any institutional affiliations.

## Supporting information


**Fig. S1** Expression of the momilactone biosynthetic orthologues within and outside the MBGC in *Oryza officinalis* and *Oryza coarctata*.
**Fig. S2** Phylogenetic analysis based on CPS4 amino acid sequence.
**Fig. S3** Phylogenetic analysis based on KSL4 amino acid sequence.
**Fig. S4** Phylogenetic analysis based on MAS amino acid sequences.
**Fig. S5** Phylogenetic analysis based on CYP99A amino acid sequences.
**Fig. S6** Microsynteny of the MBGC between *Oryza sativa* and species and sub‐genomes lacking a MBGC.
**Fig. S7** Detection of momilactone A and B in different accessions of *Oryza officinalis*.
**Fig. S8** Amino acid sequence alignment.
**Fig. S9** Prevalence of the nonsense G‐to‐A polymorphism in OoKSL4‐2 cDNA clones at the population level.
**Fig. S10** Mass spectra of peaks identified.
**Fig. S11** Synteny between *Oryza coarctata* sub‐genome LL and *Echinochloa crus‐galli* sub‐genome CC showing the lack of synteny between the genomic region containing the MBGC in *O. coarctata* (Chr4) and *E. crus‐galli* (Chr4 sub‐genome CH) at different magnifications.
**Fig. S12** Phylogenetic relationship between the *Poaceae* species used in this study.
**Fig. S13** Microsynteny analysis of the genomic regions containing the MBGC‐like.
**Fig. S14** Cladogram representing the amino acid sequence maximum‐likelihood tree with the best‐fit model selected by ModelFinder representing the relationship between the orthogroup containing *Oryza coarctata* CYP76L11.
**Fig. S15** Phylogenetic tree of the CYP76M clade from the cladogram in Fig. S14.
**Table S1** Key statistics on the reference genome assemblies used in this study.
**Table S2** Scaffold and positional information on the orthologues of MBGC genes shown in Fig. 1.
Please note: Wiley is not responsible for the content or functionality of any Supporting Information supplied by the authors. Any queries (other than missing material) should be directed to the *New Phytologist* Central Office.

## Data Availability

Code used for data analysis is available at https://github.com/spriego/Priego‐Cubero‐et‐al.‐Oryza.
