# Supplementary material for: Evolution and diversification of the momilactone biosynthetic gene cluster in the genus Oryza
Source: New Phytol. 2025 Jan 30;245(6):2681–97. doi: 10.1111/nph.20416 (PMC11840401; doi:10.1111/nph.20416)
Supplement: Supplementary file 1 — Fig. S1 Expression of the momilactone biosynthetic orthologues within and outside the MBGC in Oryza officinalis and Oryza coarctata. Fig. S2 Phylogenetic analysis based on CPS4 amino acid sequence. Fig. S3 Phylogenetic analysis based on KSL4 amino acid sequence. Fig. S4 Phylogenetic analysis based on MAS amino acid sequences. Fig. S5 Phylogenetic analysis based on CYP99A amino acid sequences. Fig. S6 Microsynteny of the MBGC between Oryza sativa and species and sub‐genomes lacking a MBGC. Fig. S7 Detection of momilactone A and B in different accessions of Oryza officinalis. Fig. S8 Amino acid sequence alignment. Fig. S9 Prevalence of the nonsense G‐to‐A polymorphism in OoKSL4‐2 cDNA clones at the population level. Fig. S10 Mass spectra of peaks identified. Fig. S11 Synteny between Oryza coarctata sub‐genome LL and Echinochloa crus‐galli sub‐genome CC showing the lack of synteny between the genomic region containing the MBGC in O. coarctata (Chr4) and E. crus‐galli (Chr4 sub‐genome CH) at different magnifications. Fig. S12 Phylogenetic relationship between the Poaceae species used in this study. Fig. S13 Microsynteny analysis of the genomic regions containing the MBGC‐like. Fig. S14 Cladogram representing the amino acid sequence maximum‐likelihood tree with the best‐fit model selected by ModelFinder representing the relationship between the orthogroup containing Oryza coarctata CYP76L11. Fig. S15 Phylogenetic tree of the CYP76M clade from the cladogram in Fig. S14. Table S1 Key statistics on the reference genome assemblies used in this study. Table S2 Scaffold and positional information on the orthologues of MBGC genes shown in Fig. 1. Please note: Wiley is not responsible for the content or functionality of any Supporting Information supplied by the authors. Any queries (other than missing material) should be directed to the New Phytologist Central Office. [file NPH-245-2681-s001.pdf]

New Phytologist Supporting Information

Article title: Evolution and diversification of the momilactone biosynthetic gene cluster in the genus *Oryza*

Authors: Santiago Priego-Cubero, Youming Liu, Tomonobu Toyomasu, Michael Gigl, Yuto Hasegawa, Hideaki Nojiri, Corinna Dawid, Kazunori Okada, Claude Becker

Article acceptance date: 31 December 2024

Figures

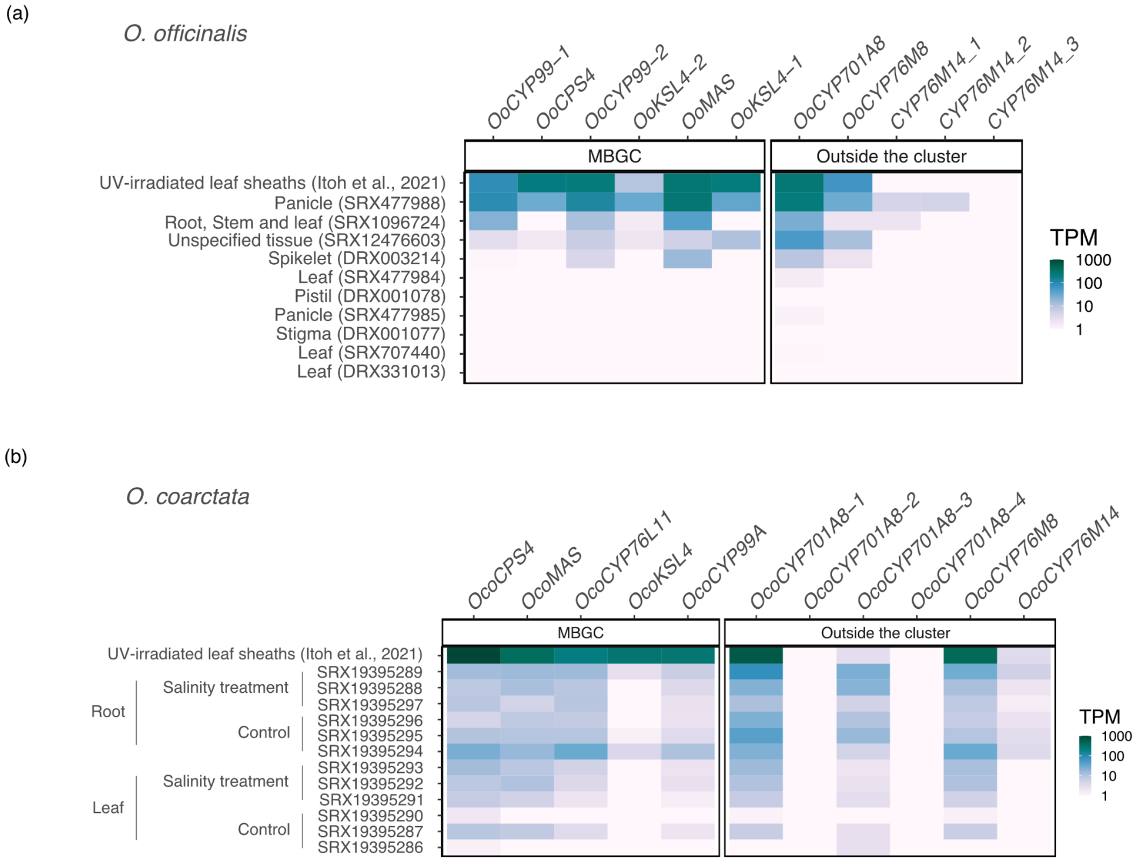

Figure S1. Expression of the momilactone biosynthetic orthologs within and outside the MBGC in a) *O. officinalis* and b) *O. coarctata*. The expression is represented as transcripts per million (TPM) and the colour is in logarithmic scale. The RNA-seq datasets were publicly available RNA-seq (see y-axis on each heatmap) and these cover different tissues and treatments. Notice that the genes belonging to the MBGC are represented in the same order as in the cluster.

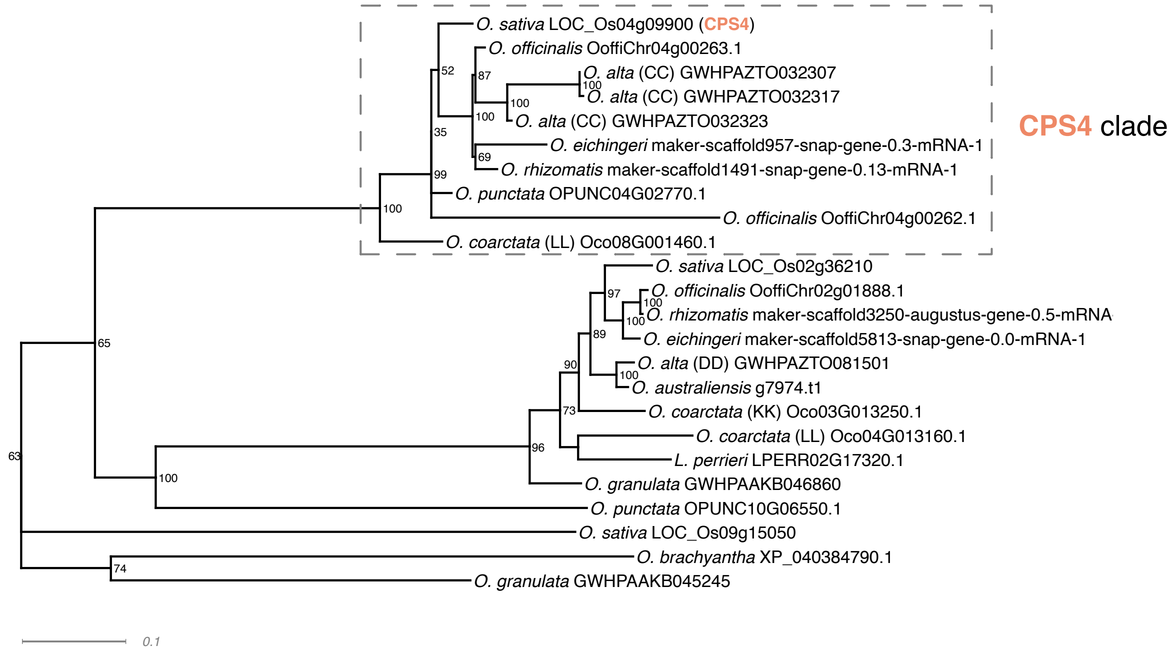

Figure S2. Phylogenetic analysis based on CPS4 amino acid sequence. Maximum likelihood tree with the best-fit model selected by ModelFinder for the orthogroup containing *O. sativa* CPS4 (LOC_Os04g09900) in *O. sativa*, *O. punctata*, *O. officinalis*, *O. eichingeri*, *O. rhizomatis*, *O. alta*, *O. australiensis*, *O. coarctata*, *O. brachyantha*, *O. granulata* and *L. perrieri*. Numbers represent 1000 replicates of ultrafast bootstrapping. Dashed rectangle highlights the CPS4 clade, which contains orthologs potentially involved in momilactones biosynthesis and clustered in *Oryza* species.

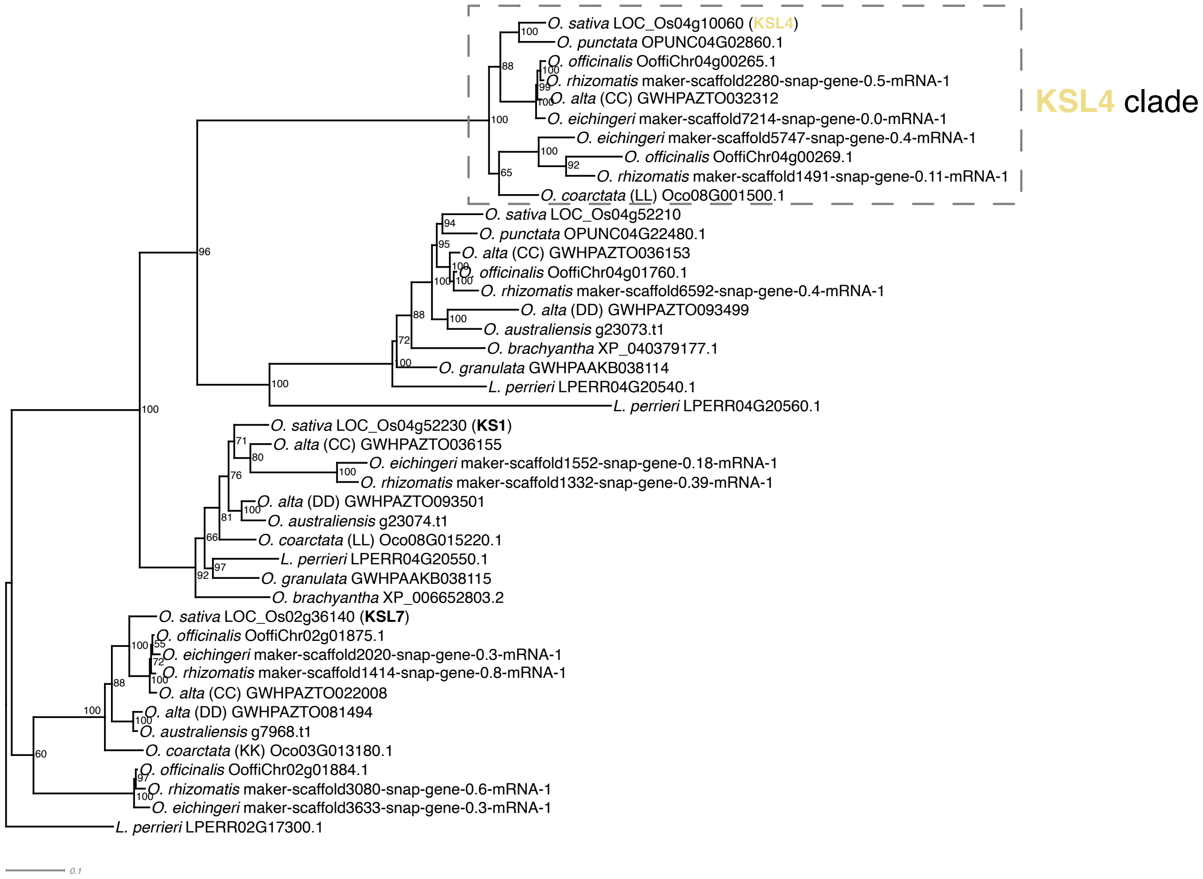

Figure S3. Phylogenetic analysis based on KSL4 amino acid sequence. Maximum likelihood tree with the best-fit model selected by ModelFinder for the orthogroup containing *O. sativa* KSL4 (LOC_Os04g10060) in *O. sativa*, *O. punctata*, *O. officinalis*, *O. eichingeri*, *O. rhizomatis*, *O. alta*, *O. australiensis*, *O. coarctata*, *O. brachyantha*, *O. granulata* and *L. perrieri*. Numbers represent 1000 replicates of ultrafast bootstrapping. Dashed rectangle highlights the KSL4 clade, which contains orthologs potentially involved in momilactones biosynthesis and clustered in *Oryza* species.

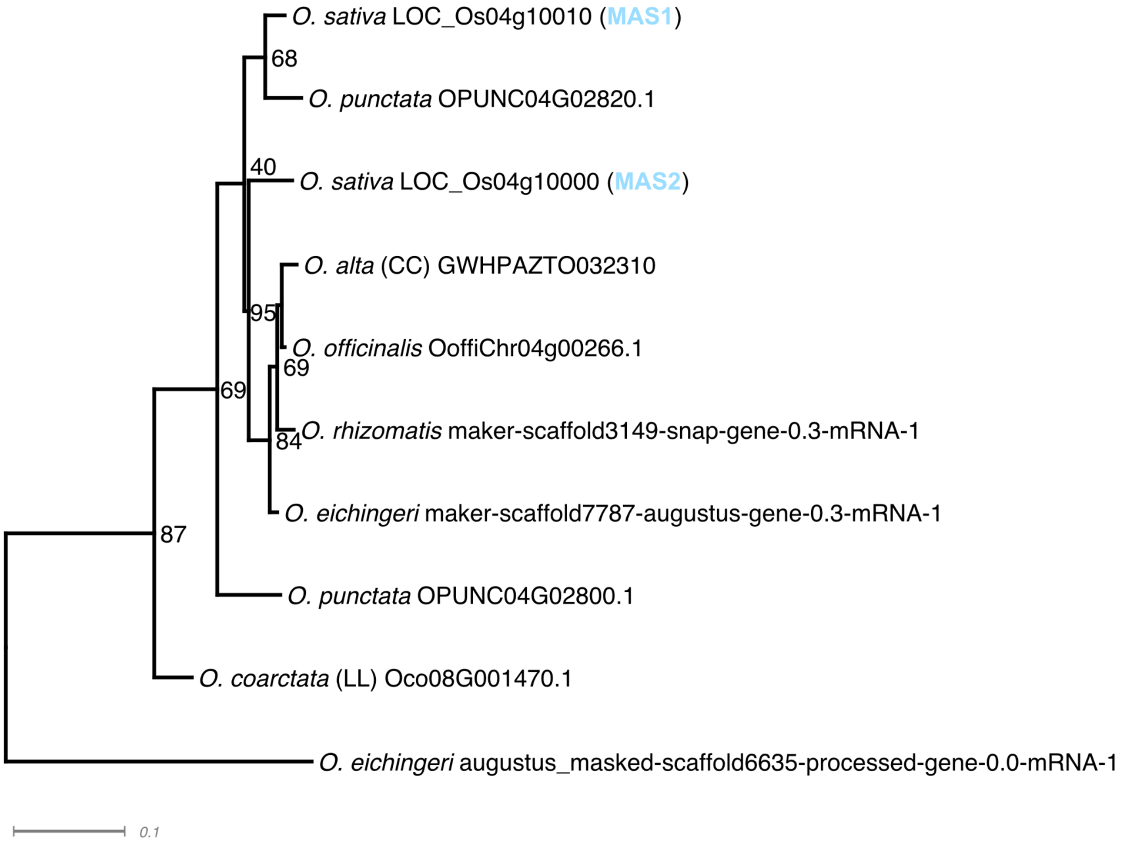

Figure S4. Phylogenetic analysis based on MAS amino acid sequences. Maximum likelihood tree with the best-fit model selected by ModelFinder for the orthogroup containing *O. sativa* MAS1 and MAS2 (LOC_Os04g10010, LOC_Os04g10000) in *O. sativa*, *O. punctata*, *O. officinalis*, *O. eichingeri*, *O. rhizomatis*, *O. alta*, *O. australiensis*, *O. coarctata*, *O. brachyantha*, *O. granulata* and *L. perrieri*. Numbers represent 1000 replicates of ultrafast bootstrapping. Since this orthogroup consisted of a small number of genes, we considered all of them as potentially involved in momilactone biosynthesis and clustered.

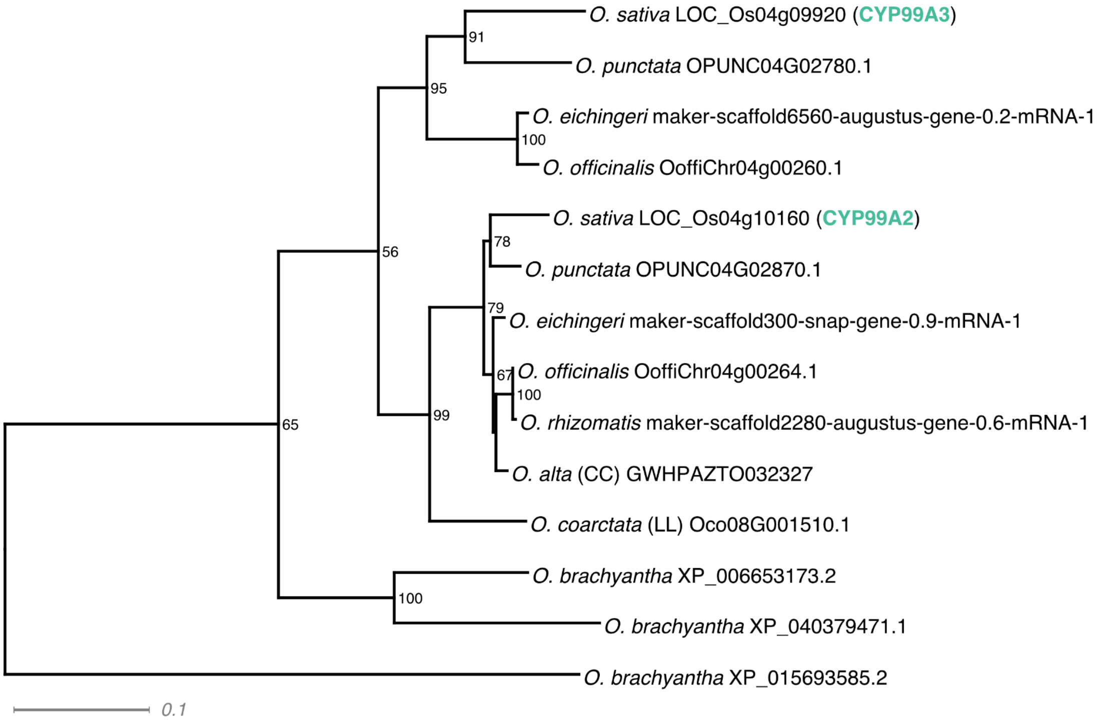

Figure S5. Phylogenetic analysis based on CYP99A amino acid sequences. Maximum likelihood tree with the best-fit model selected by ModelFinder for the orthogroup containing *O. sativa* CYP99A2 and CYP99A3 (LOC_Os04g10160, LOC_Os04g09920) in *O. sativa*, *O. punctata*, *O. officinalis*, *O. eichingeri*, *O. rhizomatis*, *O. alta*, *O. australiensis*, *O. coarctata*, *O. brachyantha*, *O. granulata* and *L. perrieri*. Numbers represent 1000 replicates of ultrafast bootstrapping. Since this orthogroup consisted of a small number of genes, we considered all of them as potentially involved in momilactone biosynthesis and clustered.

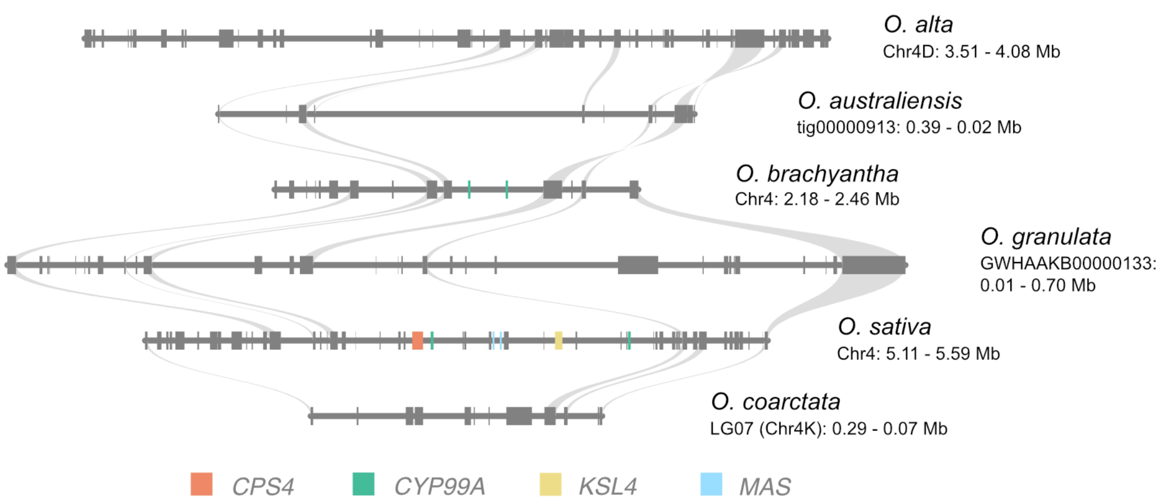

Figure S6. Microsynteny of the MBGC between *O. sativa* and species and sub-genomes lacking a MBGC. *O. alta* sub-genome DD was used as anchor species. The region was not further expanded given the fragmented nature of the assemblies for *O. australiensis* and *O. granulata*. Coloured rectangles represent the momilactone biosynthetic genes. Grey lines connect orthologs.

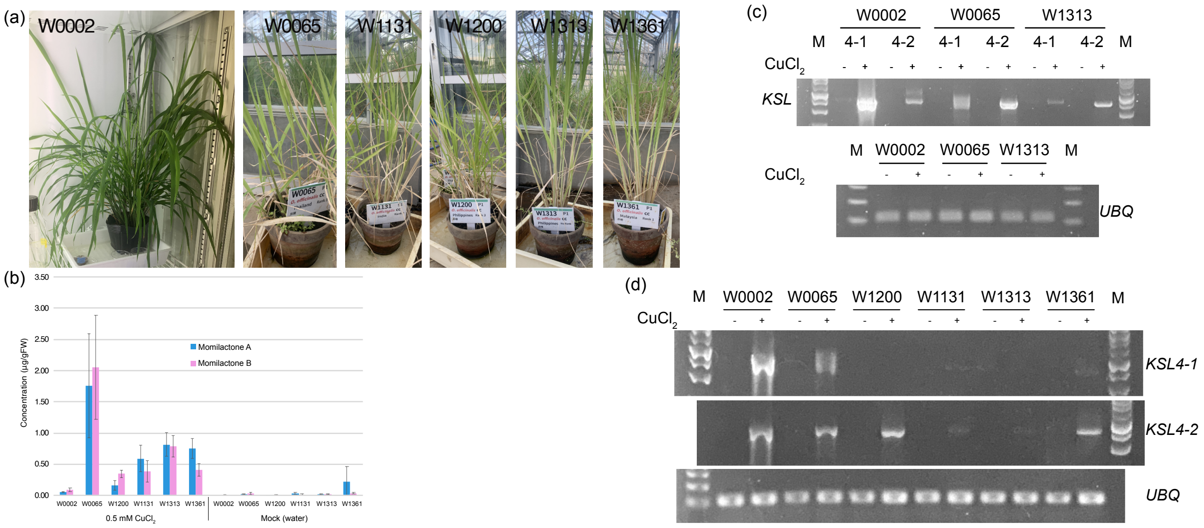

Figure S7. Detection of momilactone A and B in different accessions of *O. officinalis*. a) Accessions at the time of tissue sampling. **b)** Extracts from *O. officinalis* $CuCl_2$-treated leaf blades were analysed by liquid chromatography-tandem mass spectrometry (LC-MS/MS). Five µl of the extract were subjected to LC-MS/MS under the conditions described in the Materials and Methods section. Momilactones were detected with combinations of m/z 315/271 for momilactone A and m/z 331/269 for momilactone B in the multiple reaction monitoring mode. The reference strain, W0002, contained a premature stop codon mutation in KSL4-2, presumably resulting in low levels of momilactone production. Error bars represent standard deviation from the mean (n=3). **c)** and **d)** Reverse-transcription (RT) PCR of full-length *OoKSL4-1* and *OoKSL4-2* mRNA in different *O. officinalis* accessions under non-inducing (minus $CuCl_2$) or inducing (plus $CuCl_2$) conditions. Plants were treated with $CuCl_2$ for 72 h before RNA extraction. *UBIQUITIN* (*UBQ*) was used as a reference. **c)** Treatment and RT-PCR on a subset of lines shown in a). **d)** Independent treatment and RT-PCR on the full set of lines shown in a). Only the W0002, which carries a premature stop codon in *OoKSL4-2*, showed higher inducibility of *KSL4-1* compared to *KSL4-2*, while in all other lines, *KSL4-2* responded more strongly to $CuCl_2$.

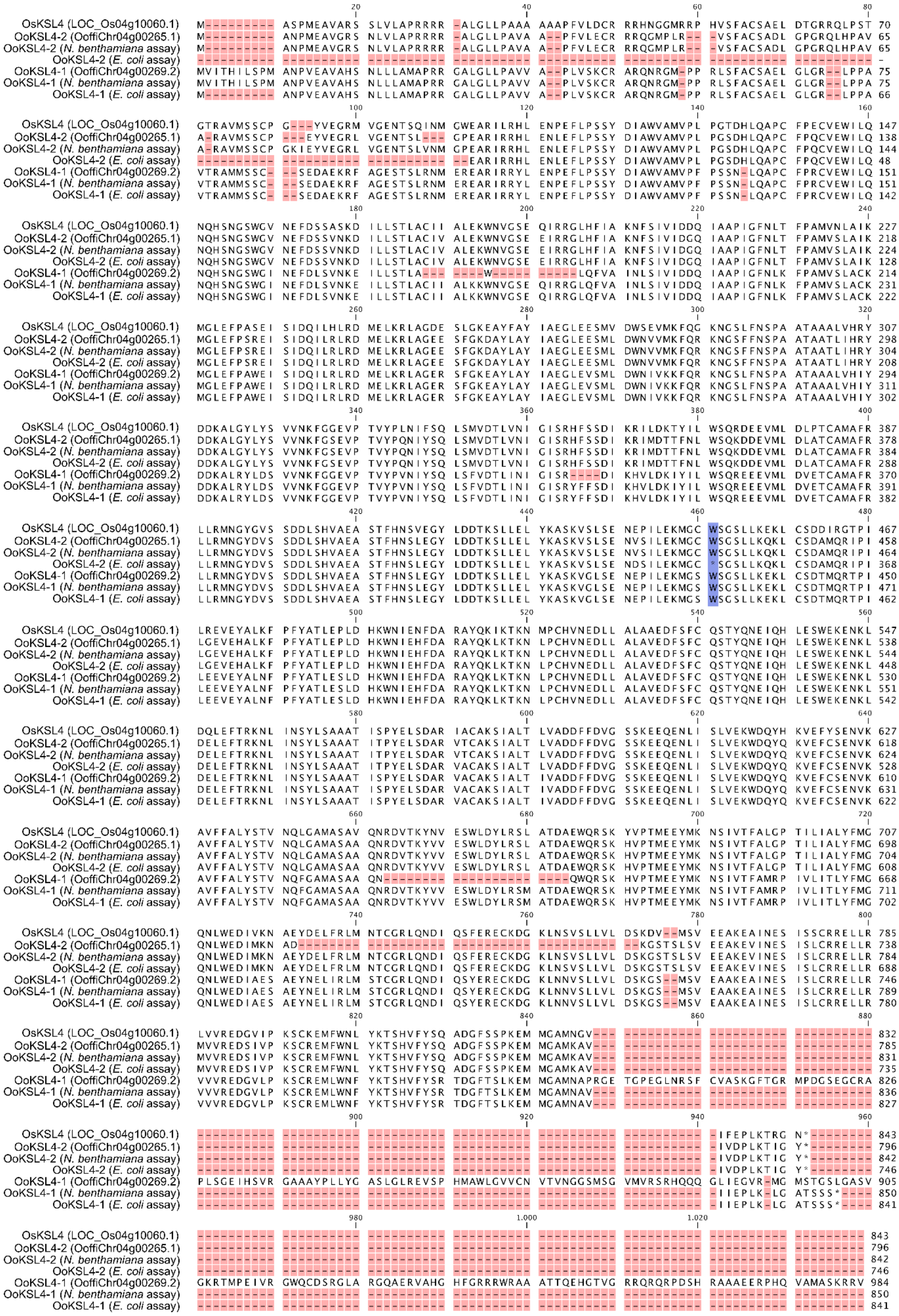

Figure S8. Amino acid sequence alignment of KSL4 orthologs. The alignment includes KSL4 from *O. sativa* (OsKSL4), its orthologs in *O. officinalis* from the annotation (KSL4-2: OoffiChr04g00265.1; KSL4-1: OoffiChr04g00269.2) and the cloned sequences used in *N. benthamiana* and *E. coli* assays. The position of the W445 clonal *de novo* mutation is highlighted in OoKSL4-2 (position assigned using the full length *OoKSL4-2* sequence cloned for the *N. benthamiana* transient expression as the reference sequence).

(a)

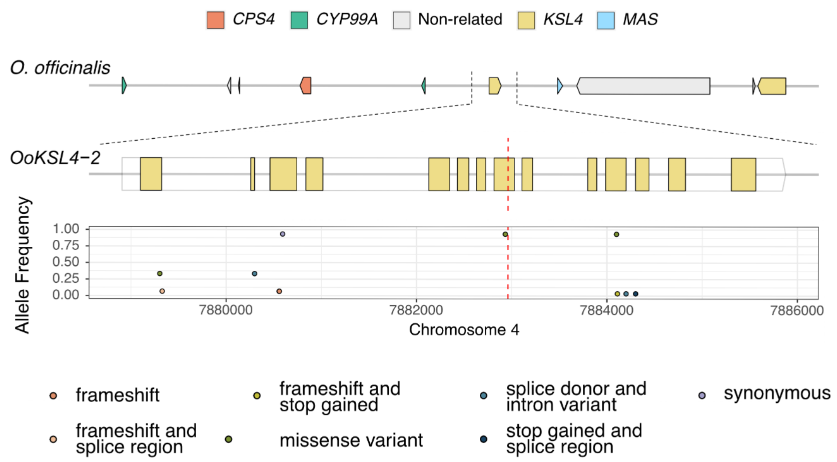

(b)

```
OoKSL4-2-W0566      TCAGTTTATCAGAAAATGACTCAATCCTAGAGAAAATGGGTTGTTGGTCAGGTAGCTTAT
OoKSL4-2-W0614      TCAGTTTATCAGAAAATGACCCAATCCTAGAGAAAATGGGTTGTTGGTCAGGTAGATTAT
OoKSL4-2-W1200      TCAGTTTATCAGAAAATGACTCAATCCTAGAGAAAATGGGTTGTTGGTCAGGTAGCTTAT
OoKSL4-2-W0002      TCAGTTTATCAGAAAATGTCTCAATCCTAGAGAAAATGGGTTGTTGGTCAGGTAGCTTAT
OoKSL4-2-W1252      TCAGTTTATCAGAAAATGACTCAATCCTAGAGAAAATGGGTTGTTGGTCAGGTAGCTTAT
OoKSL4-2-W1131      TCAGTTTATCAGAAAATGACTCAATCCTAGAGAAAATGGGTTGTTGGTCAGGTAGCTTAT
OoKSL4-2_cDNA       TCAGTTTATCAGAAAATGACTCAATCCTAGAGAAAATGGGTTGTTAGTCAGGTAGCTTAT
                      ****************** * ************************ ********* ****

OoKSL4-2-W0566      TGAAACAAAAATTGTGCTCCGATGCCATGCAAAGAATACCAATCCTTGGAGAGGTGCGTG
OoKSL4-2-W0614      TGAAACAAAAATTGTGCTCCGATGCCATGCAAAGAATACCAATCCTTGGTGAGGTGCGTG
OoKSL4-2-W1200      TGAAACAAAAATTGTGCTCCGATGCCATGCAAAGAATACCAATCCTTGGAGAGGTGCGTG
OoKSL4-2-W0002      TGAAACAAAAATTGTGCTCCGATGCCATGCAAAGAATACCAATCCTTGGAGAGGTGCGTG
OoKSL4-2-W1252      TGAAACAAAAATTGTGCTCCGATGCCATGCAAAGAATACCAATCCTTGGAGAGGTGCGTG
OoKSL4-2-W1131      TGAAACAAAAATTGTGCTCCGATGCCATGCAAAGAATACCAATCCTTGGAGAGGTGCGTG
OoKSL4-2_cDNA       TGAAACAAAAATTGTGCTCCGATGCCATGCAAAGAATACCAATCCTTGGAGAG-------
                      ************************************************** ***

OoKSL4-2-W0566      CATGAAACTCATGATCCCCATTTCAGTTGAAATTTATGTTTACATTCGATTCAGTTGGTT
OoKSL4-2-W0614      CATGAAACTCATGATCCCCATTTCAGTTGAAATTTATGTTTACATTCGATTCAGTTAGTT
OoKSL4-2-W1200      CATGAAACTCATGATCCCCATTTCAGTTGAAATTTATGTTTACATTCGATTCAGTTGGTT
OoKSL4-2-W0002      CATGAAACTCATGATCCCCATTTCAGTTGAAATTTATGTTTACATTCGATTCAGTTGGTT
OoKSL4-2-W1252      CATGAAACTCATGATCCCCATTTCAGTTGAAATTTATGTTTACATTCGATTCAGTTGGTT
OoKSL4-2-W1131      CATGAAACTCATGATCCCCATTTCAGTTGAAATTTATGTTTACATTCGATTCAGTTGGTT
OoKSL4-2_cDNA       ------------------------------------------------------------

OoffKSL4-2-W0566      TTTCTCATTTCAGGTCGAACATGCTCTCAAATTTCCATTTTATGCCACGCTT
OoffKSL4-2-W0614      TTTCTCATTTCAGGTAGAACATGCTCTCAAATTTCCATTTTATGCCACGCTT
OoffKSL4-2-W1200      TTTCTCATTTCAGGTCGAACATGCTCTCAAATTTCCATTTTATGCCACGCTT
OoffKSL4-2-W0002      TTTCTCATTTCAGGTCGAACATGCTCTCAAATTTCCATTTTATGCCACGCTT
OoffKSL4-2-W1252      TTTCTCATTTCAGGTCGAACATGCTCTCAAATTTCCATTTTATGCCACGCTT
OoffKSL4-2-W1131      TTTCTCATTTCAGGTCGAACATGCTCTCAAATTTCCATTTTATGCCACGCTT
OoffKSL4-2_cDNA       -------------GTCGAACATGCTCTCAAATTTCCATTTTATGCCACGCTT
                                   ** *************************************
```

Figure S9. Prevalence of the nonsense G-to-A polymorphism in *OoKSL4-2* cDNA clones at the population level. a) The biosynthetic gene cluster (MBGC) from *O. officinalis*. Middle panel shows a magnification of the *OoKSL4-2* locus where the yellow filled rectangles represent exons of *OoKSL4-2*. Bottom panel shows the allele frequency of single nucleotide polymorphisms (SNPs) with a potential disruptive effect on *OoKSL4-2* from 15 *O. officinalis* accessions. The SNPs represented have allele frequency > 0.2 or a putative impact annotated as 'high'. The red dashed line indicates the position of the G-to-A transition in the original cDNA clone, which is absent at the population level. **b)** Comparison of genome sequences of several *O. officinalis* accessions. The underlined codon indicates the mutation detected in the cDNA clones of OoKSL4-2 (W0002), which is absent in other accessions and in a different W0002 batch.

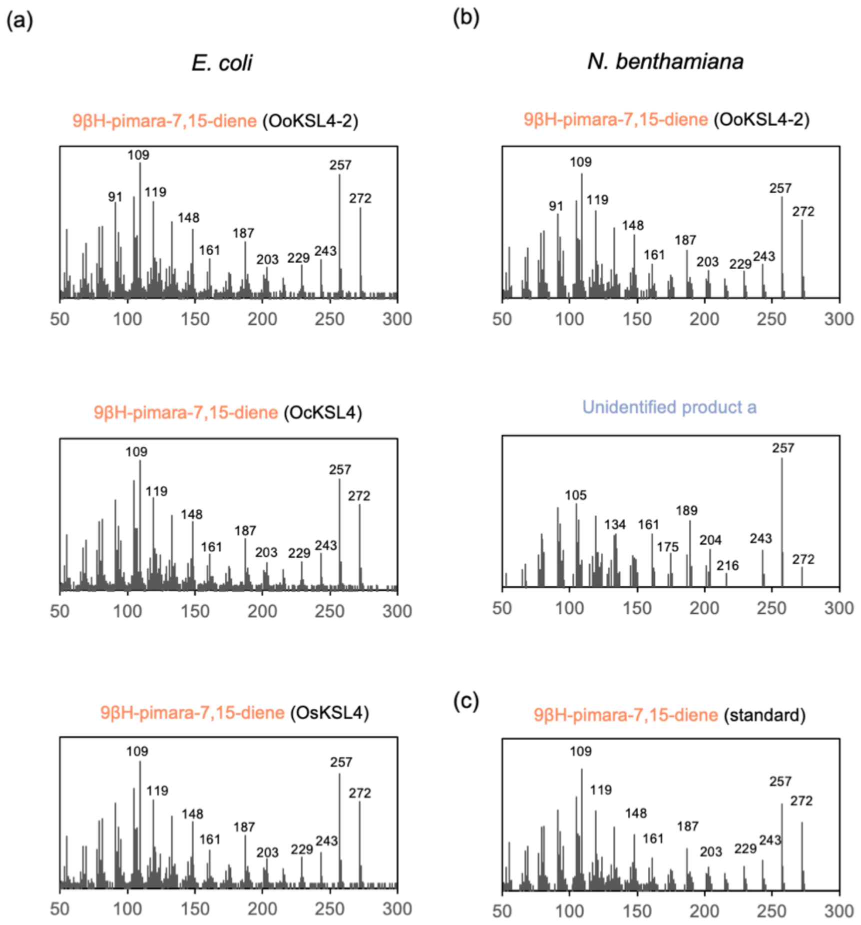

Figure S10. Mass spectra of peaks identified in *E. coli* metabolic engineering system (**a)** and *Nicotiana benthamiana* transient expression system **(b)** and (**c)** the standard of 9βH-pimara-7,15-diene.

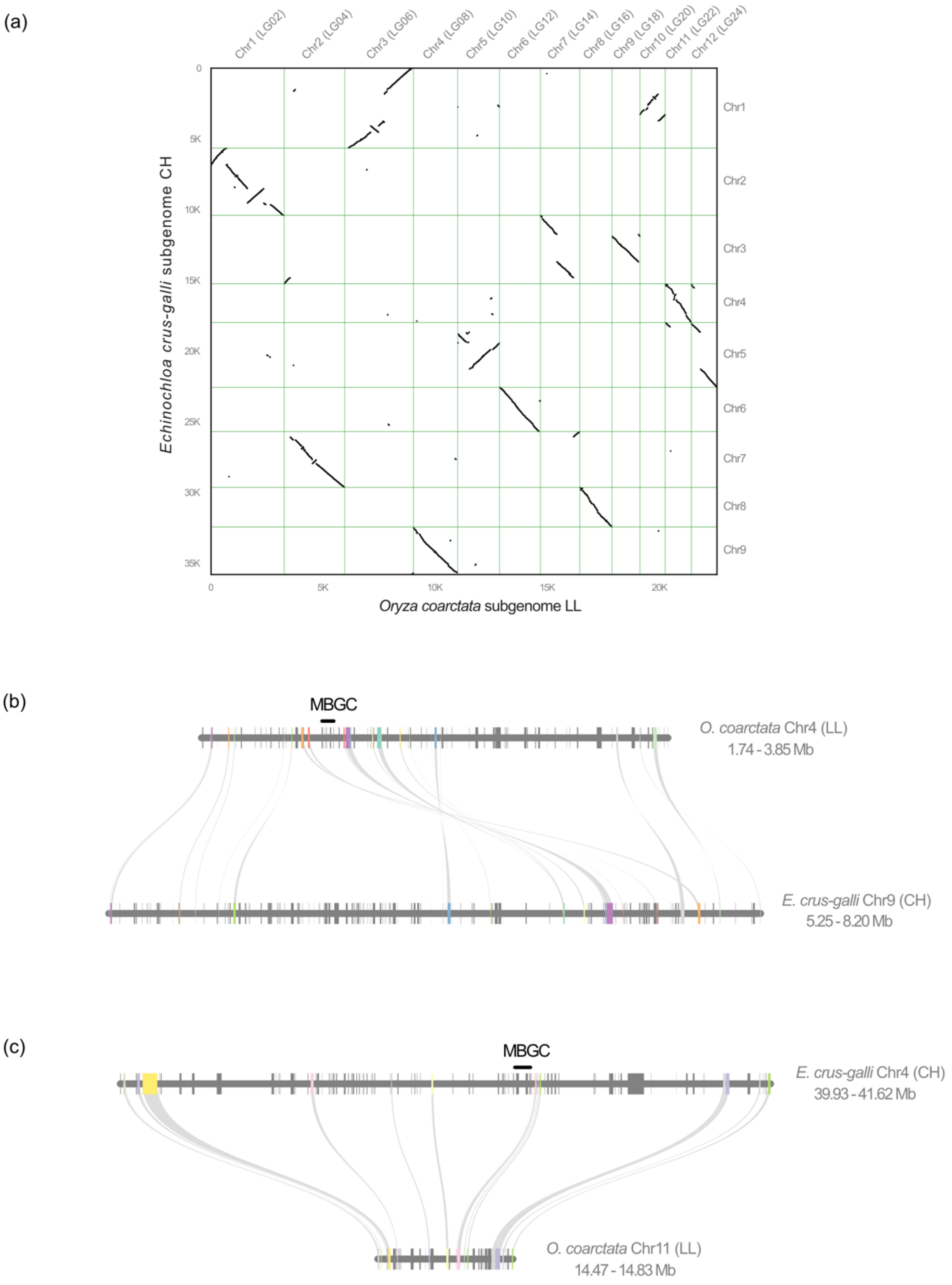

Figure S11. Synteny between *O. coarctata* sub-genome LL and *E. crus-galli* sub-genome CH showing the lack of synteny between the genomic region containing the MBGC in *O. coarctata* (Chr4) and *E. crus-galli* (Chr4 sub-genome CH). a) Dot plot showing whole-genome pairwise synteny between *O. coarctata* sub-genome LL and *E. crus-galli* sub-genome CH **b)** Synteny between the genomic region of *O. coarctata* containing MBGC (Chr4 LL) and its syntenic block in *E. crus-galli* sub-genome CH (Chr9). **c)** Synteny between the genomic region of *E. crus-galli* containing MBGC (Chr4 CH) and its syntenic block in *O. coarctata* sub-genome LL (Chr11). In (**b)** and **(c)**, grey lines connect the respective orthologs, and the black horizontal line indicates where the MBGC is located on each species.

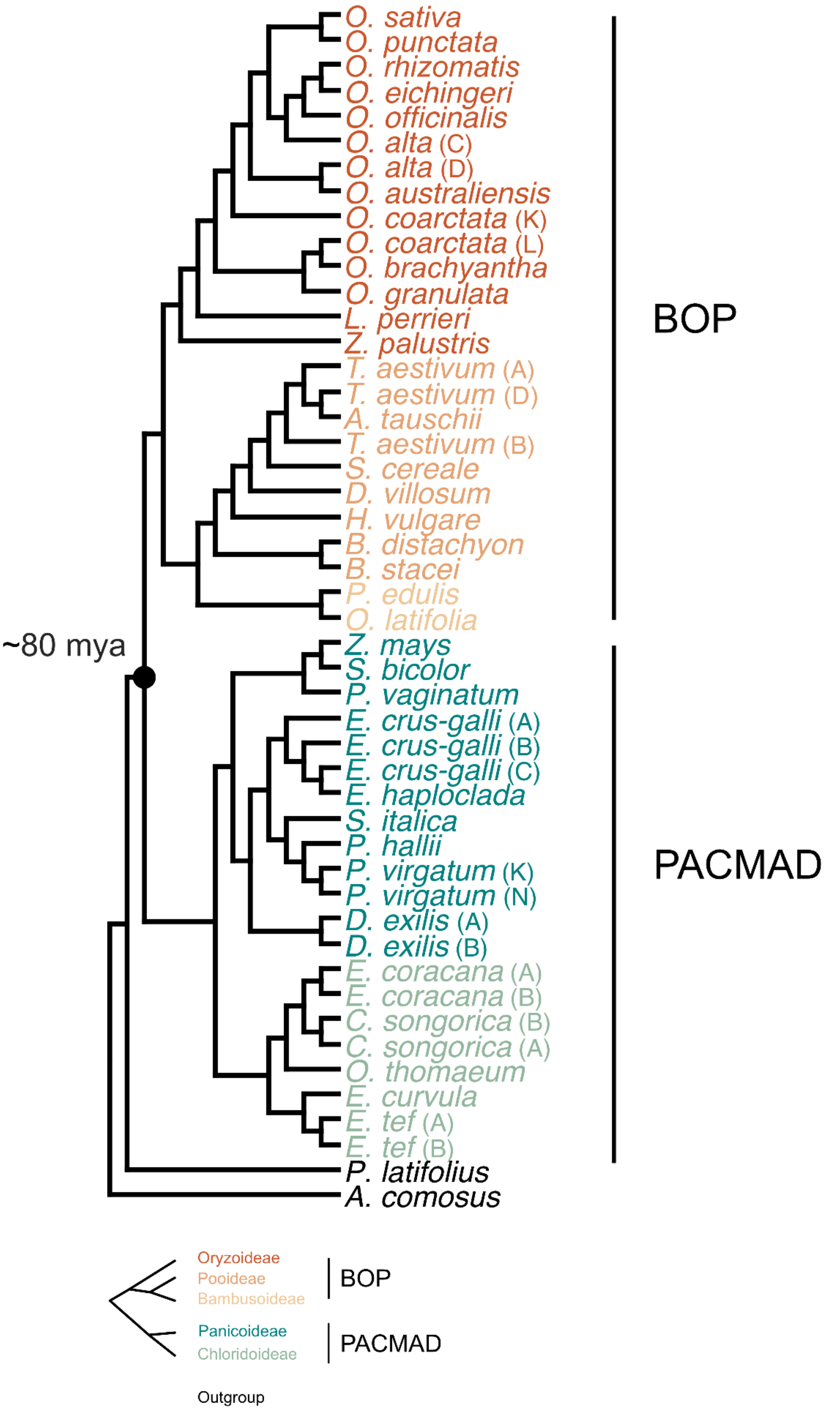

Figure S12. Phylogenetic relationship between the Poaceae species used in this study. The tree was inferred with the OrthoFinder algorithm STAG (Emms and Kelly, 2018) that uses all the species tree inferred from all orthogroups. *P. latifolius* (Poaceae) outgroup of core Poaceae (BOP and PACMAD), *A. comosus*, outgroup of Poaceae.

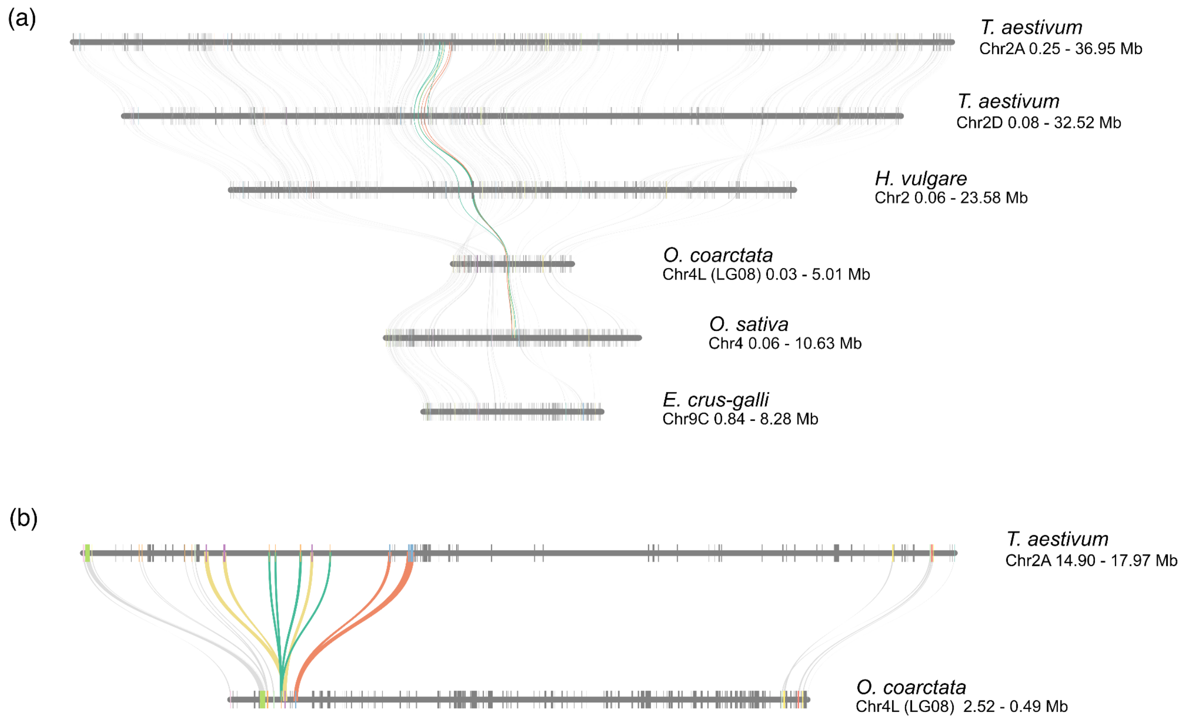

Figure S13. Microsynteny analysis of the genomic regions containing the MBGC-like from **a)** *Triticum aestivum* (wheat), *Hordeum vulgare* and the MBGC from *O. coarctata* and *O. sativa*. **b)** Magnification of the genomic region containing the MBGC-like and the MBGC in *T. aestivum* (sub-genome A) and *O. coarctata* showing the synteny between the flanking regions of both BGCs. Coloured lines represent MBGC-like orthologs and grey lines connect the respective orthologs.

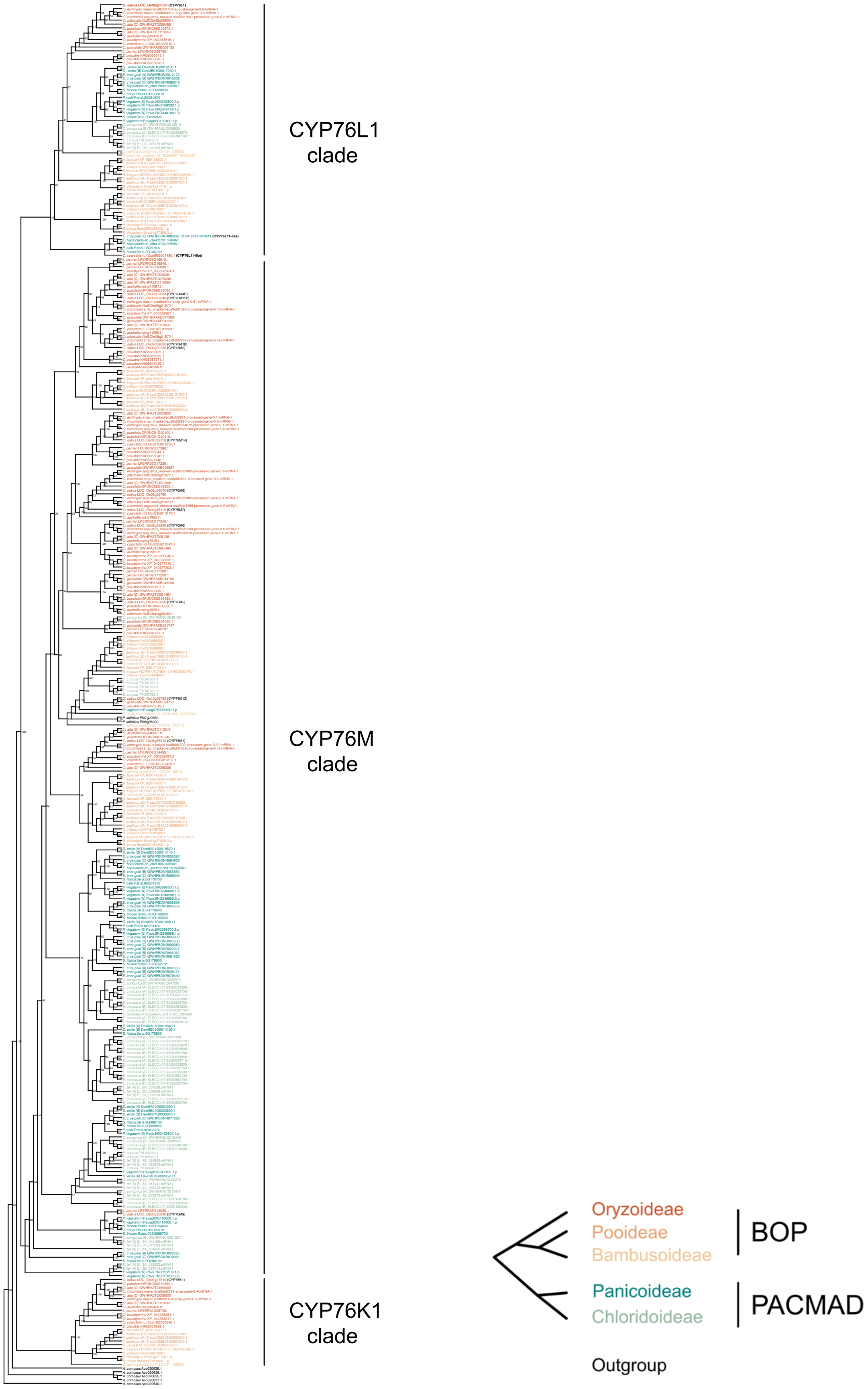
CYP76L1
clade
CYP76M
clade
CYP76K1
clade
Oryzoideae
Pooideae
Bambusoideae
BOP
Panicoideae
Chloridoideae
PACMAD
Outgroup

Figure S14 (previous page). Cladogram representing the amino-acid-sequence maximum likelihood tree with the best-fit model selected by ModelFinder representing the relationship between the orthogroup containing *O. coarctata* CYP76L11. The cladogram contains the CYP76K, CYP76L and CYP76M clades of 37 Poaceae species belonging to the subfamilies Oryzoideae, Pooideae, Bambusoideae, Panicoideae and Chloridoideae. The names of the different rice CYPs were taken from https://funricegenes.github.io/cytochrome_P450_monooxygenase_superfamily/. The phylogenetic relation between these subfamilies is represented in the bottom-right graph. Numbers represent 1000 replicates of ultrafast bootstrapping.

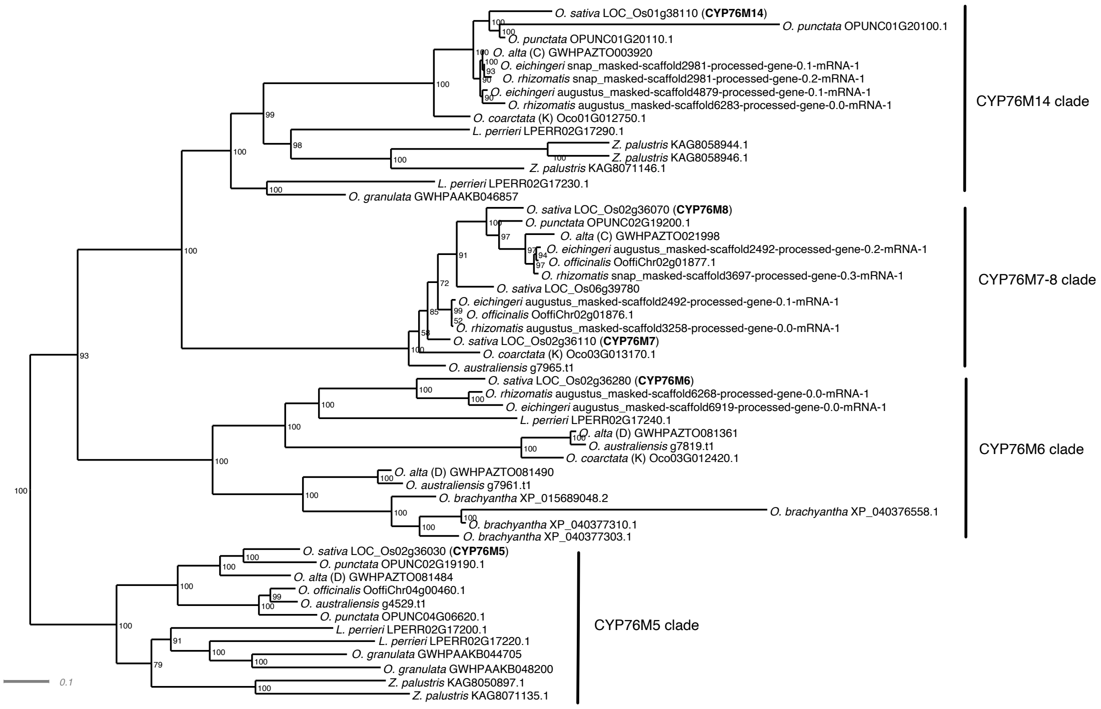

Figure S15: Phylogenetic tree of the CYP76M clade from the cladogram in Figure S14. Numbers represent 1000 replicates of ultrafast bootstrapping.

Tables

Species	Version	Genome type	Assembly Size (Mb)	Annotated Loci	Scaffold N50 (Mb)	Complete BUSCOs (%)	Reference
O. sativa	v7.0	AA	375	42189	30	95	Phytozome
O. punctata	v1.2	BB	394	31762	13	97,2	Stein et al., 2018
O. officinalis	GCA_008326285.1	CC	584	29930	0,51	94,6 or 98	Shenton et al., 2020
O. rhizomatis		CC	559	32083	0,082	90,8	Shenton et al., 2020
O. eichingeri		CC	471	3103	0,064	91,6	Shenton et al., 2020
O. alta	GWHAZTO00000000	CCDD	895	99312	37	98,2	Yu et al., 2021
		sub-genome CC	441	52861			
		sub-genome DD	435	46388			
O. australiensis	GCA_019925245.2 (MQ-UA-ANU_Oaus-KR_2.0) - Contig annotation	EE	996	51057	1,9	91,9	Phillips et al., 2022
O. coarctata			556	45571	23,1	96,22	Zhao et al., 2023
		KK	271				
		LL	261				
O. brachyantha	GCF_000231095.2 (ObraRS2)	FF	261	32038	1,6	95,9	Chen et al., 2013
O. granulata	GWHAAKB00000000	GG	737	40131	0,92	96,53	Shi et al., 2020
Leersia perrieri	v1.4	outgroup	267		8,7	97,6	Stein et al., 2018

Table S1. Key statistics on the reference genome assemblies used in this study.

Species	MBGC gene	MBGC ortholog	Chr	Orientation	Start	End	Annotation
O. sativa	CPS4	LOC_Os04g09900	Chr4	+	5318060	5326427	Phytozome
O. punctata		OPUNC04G02770	4	+	4522665	4529604	Stein et al., 2018
O. officinalis		OoffiChr04g00263	OoffiChr04	-	7769720	7775970	Shenton et al., 2020
O. eichingeri		maker-scaffold957-snap-gene-0.3	scaffold957	-	62784	69194	
O. rhizomatis		maker-scaffold1491-snap-gene-0.13	scaffold1491	-	71863	78506	
O. alta		GWHPAZTO032307 (OalC04g148840)	GWHAZTO00000004	+	4117988	4123024	Yu et al., 2021
		GWHPAZTO032317 (OalC04g148720)	GWHAZTO00000004	-	4469590	4477143	
		GWHPAZTO032323 (OalC04g148650)	GWHAZTO00000004	-	4590053	4596229	
O. coarctata		Oco08G001460	LG08	+	2283517	2288401	Zhao et al., 2023
O. sativa	KSL4	LOC_Os04g10060	Chr4	-	5428338	5434028	Phytozome
O. punctata		OPUNC04G02860	4	-	4651798	4657918	Stein et al., 2018
O. officinalis		OoffiChr04g00265 (KSL4-2)	OoffiChr04	+	7878906	7885876	Shenton et al., 2020
		OoffiChr04g00269 (KSL4-1)	OoffiChr04	-	8034073	8050226	
O. eichingeri		maker-scaffold7214-snap-gene-0.0	scaffold7214	-	6323	12766	
		maker-scaffold5747-snap-gene-0.4	scaffold5747	-	1770	5809	
O. rhizomatis		maker-scaffold2280-snap-gene-0.5	scaffold2280	-	19912	26338	
		maker-scaffold1491-snap-gene-0.11	scaffold1491	-	46688	48700	
O. alta		GWHPAZTO032312 (OalC04g148800)	GWHAZTO00000004	-	4220817	4228485	Yu et al., 2021
O. coarctata		Oco08G001500	LG08	-	2320899	2325854	Zhao et al., 2023
O. sativa	MAS	LOC_Os04g10010 (MAS1)	Chr4	-	5385722	5387215	Phytozome
		LOC_Os04g10000 (MAS2)	Chr4	-	5380095	5381565	
O. punctata		OPUNC04G02820	4	-	4619323	4620404	Stein et al., 2018
		OPUNC04G02800	4	+	4607713	4609250	
O. officinalis		OoffiChr04g00266	OoffiChr04	+	7920441	7921389	Shenton et al., 2020
O. eichingeri		maker-scaffold7787-augustus-gene-0.3	scaffold7787	-	8229	9170	
		augustus_masked-scaffold6635-processed-gene-0.0	scaffold6635	-	7865	8407	
O. rhizomatis		maker-scaffold3149-snap-gene-0.3	scaffold3149	-	49186	50134	
O. alta		GWHPAZTO032310 (OalC04g148820)	GWHAZTO00000004	-	4161740	4164110	Yu et al., 2021
O. coarctata		Oco08G001470	LG08	-	2297380	2298521	Zhao et al., 2023
O. sativa	CYP99A	LOC_Os04g09920 (CYP99A3)	Chr4	-	5332329	5334479	Phytozome
		LOC_Os04g10160 (CYP99A2)	Chr4	+	5484768	5486773	
O. punctata		OPUNC04G02780	4	-	4537911	4539644	Stein et al., 2018
		OPUNC04G02870	4	+	4692353	4694580	
O. officinalis		OoffiChr04g00260	OoffiChr04	+	7667067	7669495	Shenton et al., 2020
		OoffiChr04g00264	OoffiChr04	-	7839962	7841978	
O. eichingeri		maker-scaffold6560-augustus-gene-0.2	scaffold6560	+	5202	7197	
		maker-scaffold300-snap-gene-0.9	scaffold300	+	26448	28078	
O. rhizomatis		maker-scaffold2280-augustus-gene-0.6	scaffold2280	+	64230	65860	
O. alta		GWHPAZTO032327 (OalC04g148580)	GWHAZTO00000004	+	4644874	4645779	Yu et al., 2021
O. coarctata		Oco08G001510	LG08	+	2336194	2338065	Zhao et al., 2023
O. brachyantha		XP_006653173 (gene-LOC102721730)	NC_023166.2	-	2359703	2361535	Chen et al., 2013
O. brachyantha		XP_040379471 (gene-LOC102721444)	NC_023166.2	-	2330641	2332473	
O. sativa	CYP701A8	LOC_Os06g37300	Chr6	-	22020840	22028146	Phytozome
O. officinalis		OoffiChr06g01688	OoffiChr06	-	36504697	36512007	Shenton et al., 2020
O. coarctata		Oco11G011450 (OcCYP701A8-1)	LG11	-	16913203	16920158	Zhao et al., 2023
		Oco12G011620 (OcCYP701A8-2)	LG12	-	15919197	15926532	
		Oco12G011590 (OcCYP701A8-3)	LG12	-	15872696	15874541	
		Oco12G011610 (OcCYP701A8-4)	LG12	-	15894445	15896283	
O. sativa	CYP76M8	LOC_Os02g36070	Chr2	+	21690378	21692186	Phytozome
O. officinalis		OoffiChr02g01877	OoffiChr02g01877	-	38647795	38649297	Shenton et al., 2020
O. coarctata		Oco03G013170	LG03	+	18711059	18712555	Zhao et al., 2023
O. sativa	CYP76M14	LOC_Os01g38110	Chr1	-	21342129	21344066	Phytozome
O. officinalis		CYP76M14_1	OoffiChr01	+	31900002	31901558	This study
		CYP76M14_2	OoffiChr01	+	32851333	32852889	This study
		CYP76M14_3	OoffiChr01	+	30558655	30560211	This study
O. coarctata		Oco01G012750	LG01	-	17503624	17505405	Zhao et al., 2023

Table S2. Scaffold and positional information on the orthologs of MBGC genes shown in Figure 1.

REFERENCES

Chen, J., Huang, Q., Gao, D., Wang, Junyi, Lang, Y., Liu, T., Li, B., Bai, Z., Luis Goicoechea, J., Liang, C., Chen, C., Zhang, W., Sun, S., Liao, Y., Zhang, X., Yang, L., Song, C., Wang, M., Shi, J., Liu, G., Liu, J., Zhou, H., Zhou, W., Yu, Q., An, N., Chen, Y., Cai, Q., Wang, B., Liu, B., Min, J., Huang, Y., Wu, H., Li, Z., Zhang, Y., Yin, Y., Song, W., Jiang, J., Jackson, S.A., Wing, R.A., Wang, Jun, Chen, M., 2013. Whole-genome sequencing of *Oryza brachyantha* reveals mechanisms underlying Oryza genome evolution. Nat. Commun. 4, 1595. https://doi.org/10.1038/ncomms2596

Emms, D.M., Kelly, S., 2019. OrthoFinder: phylogenetic orthology inference for comparative genomics. Genome Biol. 20, 238. https://doi.org/10.1186/s13059-019-1832-y

Shenton, M., Kobayashi, M., Terashima, S., Ohyanagi, H., Copetti, D., Hernández-Hernández, T., Zhang, J., Ohmido, N., Fujita, M., Toyoda, A., Ikawa, H., Fujiyama, A., Furuumi, H., Miyabayashi, T., Kubo, T., Kudrna, D., Wing, R., Yano, K., Nonomura, K.-I., Sato, Y., Kurata, N., 2020. Evolution and diversity of the wild rice *Oryza officinalis* complex, across continents, genome types, and ploidy levels. Genome Biol. Evol. 12, 413–428. https://doi.org/10.1093/gbe/evaa037

Stein, J.C., Yu, Y., Copetti, D., Zwickl, D.J., Zhang, L., Zhang, C., Chougule, K., Gao, D., Iwata, A., Goicoechea, J.L., Wei, S., Wang, J., Liao, Y., Wang, M., Jacquemin, J., Becker, C., Kudrna, D., Zhang, J., Londono, C.E.M., Song, X., Lee, S., Sanchez, P., Zuccolo, A., Ammiraju, J.S.S., Talag, J., Danowitz, A., Rivera, L.F., Gschwend, A.R., Noutsos, C., Wu, C., Kao, S., Zeng, J., Wei, F., Zhao, Q., Feng, Q., El Baidouri, M., Carpentier, M.-C., Lasserre, E., Cooke, R., Rosa Farias, D. da, da Maia, L.C., dos Santos, R.S., Nyberg, K.G., McNally, K.L., Mauleon, R., Alexandrov, N., Schmutz, J., Flowers, D., Fan, C., Weigel, D., Jena, K.K., Wicker, T., Chen, M., Han, B., Henry, R., Hsing, Y.C., Kurata, N., de Oliveira, A.C., Panaud, O., Jackson, S.A., Machado, C.A., Sanderson, M.J., Long, M., Ware, D., Wing, R.A., 2018. Genomes of 13 domesticated and wild rice relatives highlight genetic conservation, turnover and innovation across the genus *Oryza*. Nat. Genet. 50, 285–296. https://doi.org/10.1038/s41588-018-0040-0

Yu, H., Lin, T., Meng, X., Du, H., Zhang, J., Liu, G., Chen, Mingjiang, Jing, Y., Kou, L., Li, X., Gao, Q., Liang, Yan, Liu, X., Fan, Z., Liang, Yuntao, Cheng, Z., Chen, Mingsheng, Tian, Z., Wang, Y., Chu, C., Zuo, J., Wan, J., Qian, Q., Han, B., Zuccolo, A., Wing, R.A., Gao, C., Liang, C., Li, J., 2021. A route to de novo domestication of wild allotetraploid rice. Cell 184, 1156-1170.e14. https://doi.org/10.1016/j.cell.2021.01.013

Zhao, H., Gao, Z., Wang, L., Wang, J., Wang, Songbo, Fei, B., Chen, C., Shi, C., Liu, X., Zhang, H., Lou, Y., Chen, L., Sun, H., Zhou, X., Wang, Sining, Zhang, C., Xu, H., Li, L., Yang, Y., Wei, Y., Yang, W., Gao, Q., Yang, H., Zhao, S., Jiang, Z., 2018. Chromosome-level reference genome and alternative splicing atlas of moso bamboo (*Phyllostachys edulis*). GigaScience 7, giy115. https://doi.org/10.1093/gigascience/giy115
